# Interfacial Engineering in Rare‐Earth Oxide/ZnO Heterojunctions for High‐Performance Trimethylamine Sensing and Fish Freshness Monitoring

**DOI:** 10.1002/advs.202522490

**Published:** 2026-01-22

**Authors:** Junxi Cheng, Chang Liu, Miaomiao Liu, Qihua Sun, Jun Sun, Zhaofeng Wu

**Affiliations:** ^1^ School of Materials Science and Engineering Xinjiang University Urumqi Xinjiang China; ^2^ Xinjiang Engineering Research Center for Environmental Functional Materials Xinjiang University Urumqi Xinjiang China; ^3^ School of Physics Science and Technology Xinjiang University Urumqi Xinjiang China

**Keywords:** DFT calculation, fish freshness, interfacial engineering, rare‐earth oxide/ZnO heterojunction, trimethylamine sensor

## Abstract

Developing high‐performance gas sensors for room‐temperature (RT) operation remains a significant challenge in the field of gas detection. This study addresses this issue by designing and synthesizing a series of rare‐earth (RE) oxide modified ZnO heterojunctions (RE_2_O_3_/ZnO, RE = Nd, Y, Yb) via a co‐precipitation method. Systematic characterization combined with density functional theory (DFT) calculations reveals that the Yb_2_O_3_/ZnO heterostructure exhibits optimal morphology, abundant oxygen vacancies, and favorable band alignment. The resultant sensor demonstrates exceptional performance for trimethylamine (TMA) detection at RT, featuring a high response (6.84k% to 500 ppm), ultra‐fast response/recovery kinetics (8/14 s), excellent selectivity, and a low theoretical detection limit (0.926 ppm). The enhanced sensing mechanism is attributed to the strong Lewis acidity and high polarizability of Yb^3+^, which optimizes charge transfer and promotes TMA adsorption. Furthermore, the practical utility of the Yb_2_O_3_/ZnO sensor is successfully demonstrated through real‐time monitoring of fish spoilage, showing a strong linear correlation with storage time. This work not only presents a superior RT gas sensor but also provides deep insights into the role of RE cations in modulating heterojunction properties, offering a valuable strategy for designing advanced sensing materials.

## Graphical abstract

This study realizes superior gas sensing performance via Yb_2_O_3_/ZnO heterojunction design, achieving highly sensitive/selective trimethylamine detection at room temperature while elucidating the intrinsic sensing mechanism. This innovation is applied to reliable real‐time fish freshness monitoring, paving a feasible way for translating advanced material design to practical food quality inspection.

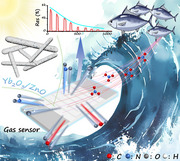

## 1 Introduction

Trimethylamine (TMA) is a common volatile organic compound (VOC) produced during food spoilage, aquaculture, and chemical manufacturing [1]. It serves as a reliable marker of seafood freshness and presents acute health hazards; sustained exposure irritates the respiratory tract and impairs neurological function, while accumulated emissions also disturb aquatic and soil systems. In clinical analysis, TMA concentrations of 0.1–0.2 ppm in exhaled breath have been established as a biomarker for kidney disease [2]. Although conventional VOC detection techniques such as GC‐MS [3] and FTIR [4] offer high sensitivity and excellent species identification capabilities, they rely on sophisticated instruments, specialized operation, and tedious sample pretreatment, which limits their application in low‐cost, on‐site, and real‐time monitoring scenarios. Metal oxide semiconductor (MOS) gas sensors are a mainstream technology for VOC detection, owing to their low cost, ease of fabrication, and compatibility with integration [5]. Conventional MOS sensors, however, typically require high operating temperatures ranging from 200°C to 400°C to activate the necessary surface reactions. This leads to significant energy consumption, limited portability, and long‐term performance degradation [6]. Therefore, the development of MOS sensors capable of operating at room temperature (RT) with high sensitivity and selectivity has emerged as a central research objective. Nevertheless, progress in this area is often constrained by low charge carrier mobility and an insufficient number of active sites under RT conditions [7].

Zinc oxide (ZnO), a representative n‐type metal oxide semiconductor, has garnered significant interest for gas sensing applications due to its favorable properties, including tunable electrical conductivity, high stability, low cost, and non‑toxicity [8]. However, ZnO‑based gas sensors often exhibit limited gas selectivity and typically require elevated operating temperatures. To address these constraints, constructing heterojunctions has become a crucial strategy. The built‑in electric field formed at the heterojunction interface promotes the separation of charge carriers, while lattice mismatch‑induced defects can enhance gas adsorption and improve the kinetics of surface reactions [9].

Among various materials for modifying ZnO, trivalent rare‑earth (RE) oxides, notably Nd_2_O_3_, Y_2_O_3_, and Yb_2_O_3_, are regarded as ideal candidates for forming heterojunctions. These oxides exhibit stable +3 valence states, high chemical inertness, and excellent structural stability even under harsh conditions, which support their broad use in catalysis, optics, and gas sensing [10, 11, 12, 13] Their ionic radii are well matched with that of Zn^2+^, promoting the formation of coherent heterointerfaces that enable efficient charge transfer. Electronically, the presence of unfilled 4f orbitals confers high polarizability, a property that is expected to enhance the selective adsorption of nitrogen‑containing molecules such as TMA [14]. Moreover, their wide bandgaps can align favorably with the energy bands of ZnO, thereby optimizing interfacial charge transfer and improving both electrical conductivity and surface reactivity [15].

While modifications of MOS with RE oxides have been studied, current research is largely focused on individual RE elements [16]. A systematic comparative study remains notably absent. This gap impedes a fundamental understanding of how different RE cations, specifically Nd^3^^+^, Y^3^^+^, and Yb^3^^+^ with their distinct 4f electron configurations, differentially modulate the charge transport properties and surface chemistry of ZnO‐based heterojunctions. As a result, the specific roles and comparative effectiveness of Nd, Y, and Yb oxides in regulating interfacial characteristics and the ensuing RT gas‐sensing mechanisms are still not fully clarified.

To address this knowledge gap, we systematically prepared and compared ZnO heterojunctions modified with three different trivalent RE oxides: Nd_2_O_3_, Y_2_O_3_, and Yb_2_O_3_. The structure–activity relationships were elucidated through meticulous microstructural characterization, comprehensive gas‑sensing evaluation, and density functional theory (DFT) calculations. Results indicate that the Yb_2_O_3_/ZnO sensor exhibits superior room‑temperature sensing performance toward TMA. This enhancement is correlated with an optimized interfacial charge transfer and, as supported by DFT calculations, the strongest TMA adsorption energy among the studied systems. Furthermore, the practical viability of the optimized sensor was successfully demonstrated via real‑time monitoring of seafood freshness, effectively connecting laboratory research with real‐world application. This work provides important insights into the selection and design principles of rare‑earth‑modified MOS sensors for high‑performance RT gas detection.

## 2 Results and Discussion

### 2.1 Morphological and Composition of Samples

**FIGURE 1:**
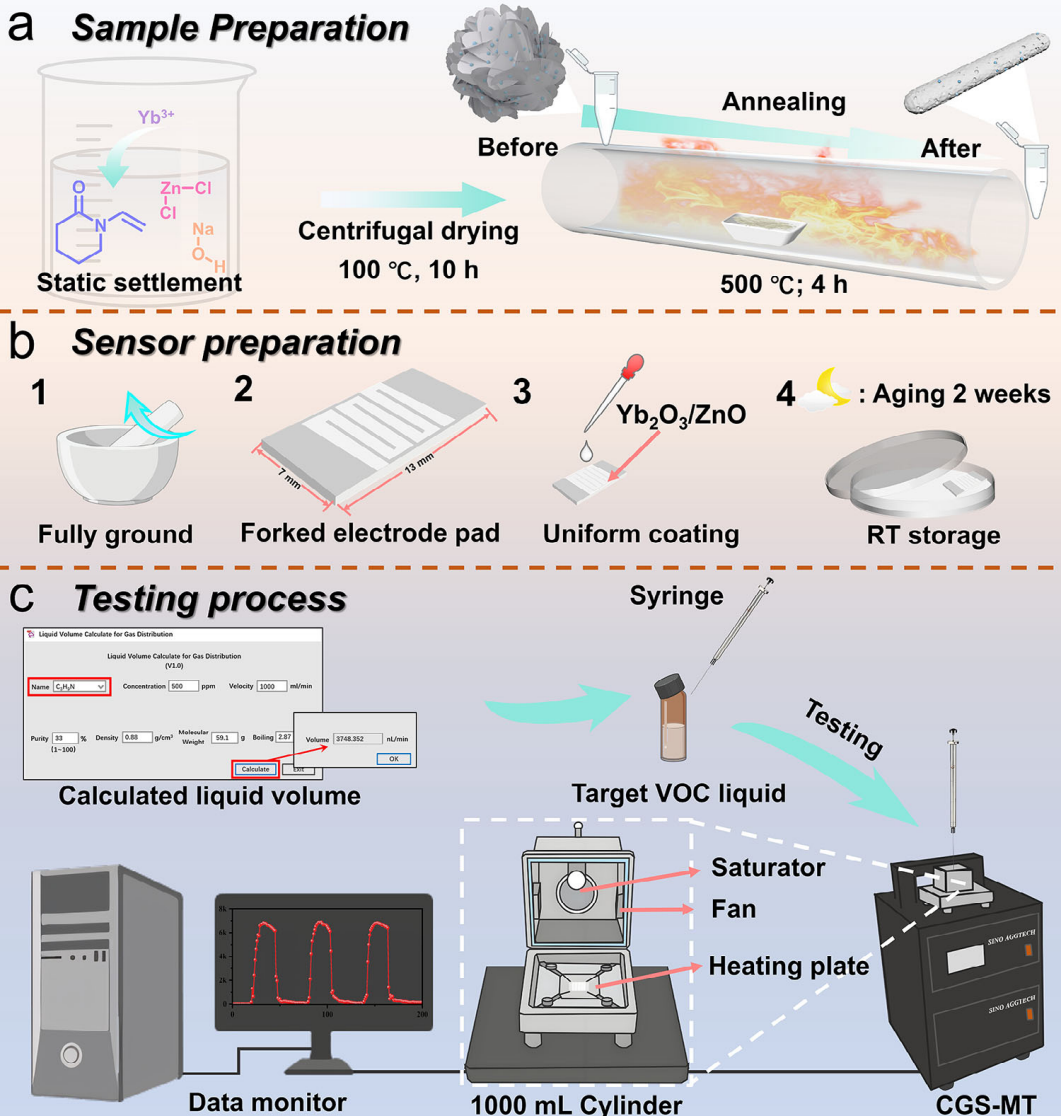
Schematic illustrations of (a) the Yb_2_O_3_/ZnO sample preparation, (b) the sensor preparation, and (c) the gas sensing testing process.

ZnO and RE_2_O_3_/ZnO (RE = Nd, Y, Yb) heterostructures were synthesized via a co‑precipitation method followed by calcination. Gas sensors were fabricated by depositing the prepared powders onto fork‑shaped interdigital electrodes. Their gas‑sensing properties toward TMA, including response, selectivity, and stability, were systematically evaluated under RT conditions (Figure 1).

**FIGURE 2:**
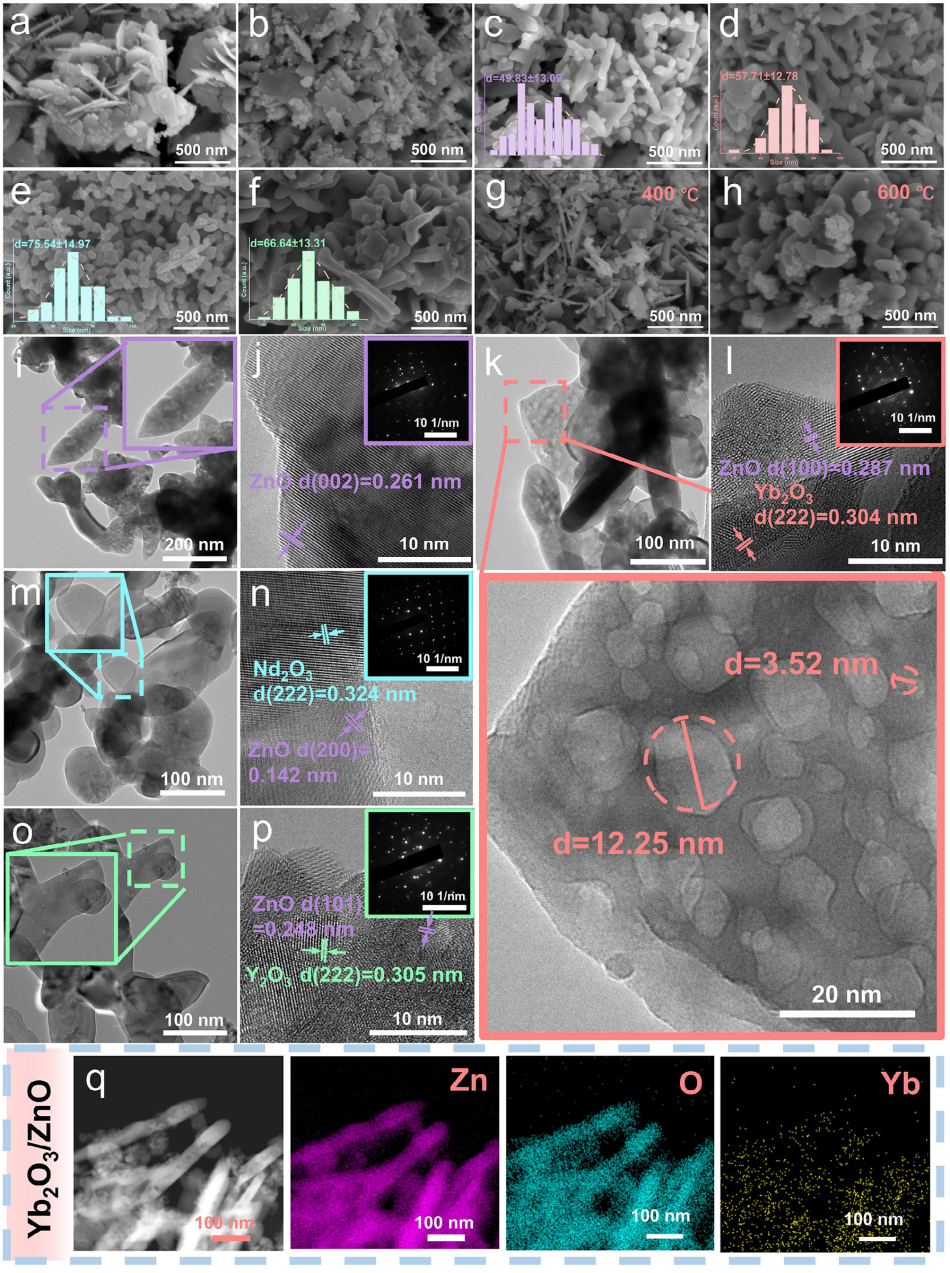
Microstructural and elemental characterization. (a,b) SEM images of the ZnO and Yb_2_O_3_/ZnO precursors; (c–f) SEM images of annealed ZnO, Yb_2_O_3_/ZnO, Nd_2_O_3_/ZnO, and Y_2_O_3_/ZnO; (g,h) SEM images of Yb_2_O_3_/ZnO annealed at 400°C and 600°C (insets: corresponding particle‐size distributions); (i,j) TEM images of ZnO; (k,l) Yb_2_O_3_/ZnO; (m,n) Nd_2_O_3_/ZnO; (o,p) Y_2_O_3_/ZnO (insets: corresponding SAED patterns); (q) EDS elemental mapping of Yb_2_O_3_/ZnO.

The microstructural evolution of the ZnO and RE_2_O_3_/ZnO (RE = Nd, Y, Yb) composites (Figure S1) before and after annealing was systematically investigated to correlate their morphology with gas‐sensing properties. As‐synthesized ZnO (Figure 2a) exhibited loosely agglomerated irregular nanosheets and needle‐like structures. In contrast, the Yb_2_O_3_/ZnO sample (Figure 2b) displayed a layered framework decorated with abundant nanoparticles, indicating that RE^3+^ incorporation altered the crystal growth pathway (Figure S2). After annealing at 500°C, ZnO transformed into irregular nanoparticles (Figure 2c), while Yb_2_O_3_/ZnO evolved into uniform rod‐like grains with an average size of 57.71 nm and abundant surface porosity (Figure 2d). In comparison, the annealed Nd_2_O_3_/ZnO and Y_2_O_3_/ZnO samples predominantly consisted of spherical or flake‐like particles with broader size distributions (Figure 2e,f). Among all composites, Yb_2_O_3_/ZnO possessed the smallest and most uniform particle size, which is beneficial for providing more active sites and enhancing gas‐solid interaction efficiency [17]. The influence of annealing temperature on the morphological evolution of Yb_2_O_3_/ZnO was further examined (Figure 2g,h). The sample annealed at 400°C retained its layered architecture but with fewer surface particles. Annealing at 600°C, however, led to severe agglomeration and dense packing, which would be expected to hinder gas adsorption‑desorption kinetics [18]. The optimal morphology, characterized by well‐dispersed rod‐like particles and rich surface porosity, was achieved after annealing at 500°C.

TEM analysis provided further structural insight. While ZnO particles showed sparse surface pores (Figure 2i,j), Yb_2_O_3_/ZnO rods exhibited abundant interconnected pores with sizes ranging from 3.52 to 12.25 nm (Figure 2k,l; Figure S3), favorable for gas diffusion, especially when compared to the microstructures of Nd_2_O_3_/ZnO (Figure 2m,n) and Y_2_O_3_/ZnO (Figure 2o,p). HRTEM imaging revealed distinct lattice fringes of 0.287 and 0.304 nm, corresponding to the (100) plane of ZnO and the (222) plane of Yb_2_O_3_, respectively, confirming the successful formation of a heterojunction. SAED patterns indicated the composite's single‐crystalline nature, and EDS mapping (Figure 2q) confirmed the homogeneous distribution of Yb within the ZnO matrix.

**FIGURE 3:**
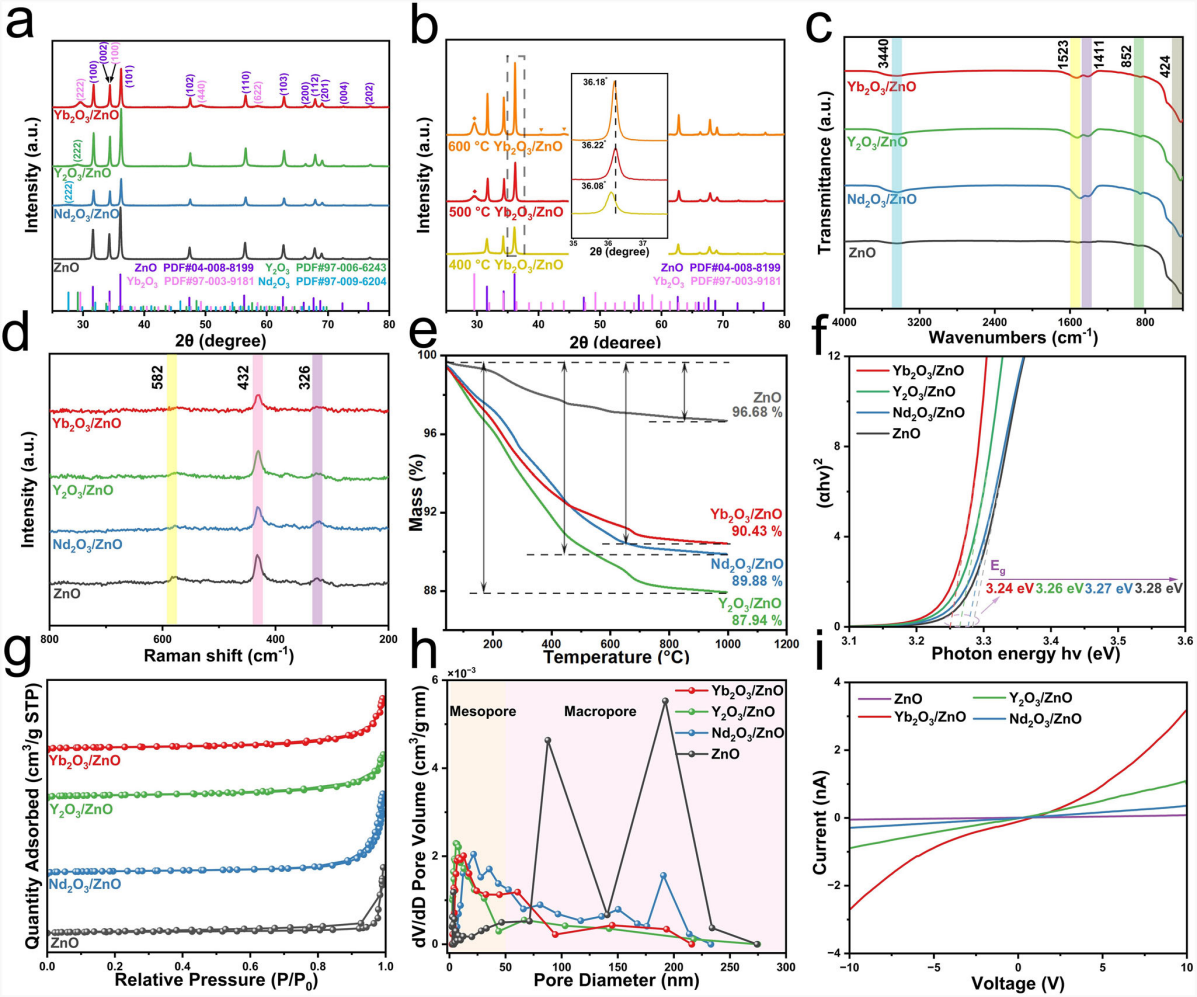
Structure‐Property Relationships in RE_2_O_3_/ZnO (RE = Nd, Y, Yb) Composites. (a) XRD patterns of ZnO and RE_2_O_3_/ZnO composites. (b) XRD patterns of Yb_2_O_3_/ZnO annealed at 400°C, 500°C, and 600°C. (c) FTIR spectra, (d) Raman spectra, (e) TGA curves, (f) Tauc plots, (g) N_2_ adsorption–desorption isotherms, (h) pore size distribution, and (i) electrical conductivity (*I*–*V* curves).

The XRD patterns of ZnO and RE_2_O_3_/ZnO (RE = Nd, Y, Yb) are shown in Figure 3a. All diffraction peaks are indexed to the hexagonal wurtzite structure of ZnO (PDF#04‐008‐8199), confirming their high crystallinity. For the Yb_2_O_3_/ZnO sample, characteristic peaks corresponding to Yb_2_O_3_ (PDF#97‐003‐9181) are observed at 29.5°, 49.3°, and 58.5°, verifying the successful formation of a heterostructure. The influence of annealing temperature was investigated (Figure 3b). The (101) diffraction peak of Yb_2_O_3_/ZnO shifts to a higher angle after annealing at 500°C, suggesting the introduction of maximized compressive strain at the heterointerface. In contrast, a shift back to a lower angle occurs at 600°C, indicating strain relaxation due to thermal effects. This evolution identifies 500°C as the optimal temperature for achieving a stable interfacial structure.

The FT‐IR spectra (Figure 3c) show a broad absorption band in the range of 3300–3500 cm^−1^, which is attributed to the O─H stretching vibrations of adsorbed water molecules [19]. A weak peak observed at 1523 cm^−^^1^ corresponds to the bending vibration of absorbed water and surface hydroxyl groups [20]. Additionally, the absorption at 424 cm^−1^ is assigned to the bending vibration of Zn─O bonds [21]. Raman spectroscopy was employed to unveil the structural characteristics and further details of ZnO and RE_2_O_3_/ZnO (RE = Nd, Y, Yb). As shown in Figure 3d, the Raman spectra of all samples exhibit characteristic sharp peaks at approximately 432 and 582 cm^−1^, which are attributed to the E_2_(high) and E_1_(LO) phonon modes of the wurtzite structure of ZnO, respectively, indicating the good crystallinity of the samples [22, 23]. The peak at ∼326 cm^−1^ corresponds to the E_2_(high)–E_2_(low) second‐order phonon mode of ZnO [24].

TGA and DSC analyses (Figure 3e; Figure S5) reveal similar thermal behavior across all samples. The total weight loss upon heating to 1000°C ranges from 3.32% to 12.06%, with Yb_2_O_3_/ZnO demonstrating the highest thermal stability. An exothermic peak near 570°C in the DSC curve corresponds to the combustion of residual organic species [25]. UV–vis spectra (Figure S6) exhibit strong absorption in the ultraviolet region. The Tauc plot (Figure 3f) confirms the direct bandgap nature of all samples. Compared with pristine ZnO (3.28 eV), every RE_2_O_3_/ZnO composite exhibits a reduced gap, most notably Yb_2_O_3_/ZnO (3.24 eV). This narrowing, induced by heterojunction formation, facilitates electron excitation and promotes carrier generation and separation [26]. Consequently, oxygen molecules adsorbed on the surface capture conduction‐band electrons more efficiently, increasing the concentration of chemisorbed oxygen and widening the depletion layer [26], which collectively enhance the gas‐sensing response [15]. XPS valence band spectra (Figure S7) reveal that the valence band maximum (VBM) shifts from 2.452 eV for pure ZnO to 2.158 eV for the Yb_2_O_3_/ZnO composite. This shift indicates a reduced energy barrier for surface reactions.

N_2_ adsorption‐desorption isotherms (Figure 3g,h) indicate that the RE_2_O_3_/ZnO heterostructures possess higher specific surface areas than pure ZnO. The Yb_2_O_3_/ZnO isotherm is type‐IV with an H3 hysteresis loop, characteristic of a mesoporous structure. Notably, Yb_2_O_3_/ZnO has the largest pore volume (0.23 cm^3^ g^−1^), beneficial for gas storage and diffusion. The pore size of RE_2_O_3_/ZnO is significantly smaller than that of pure ZnO (82.33 nm), primarily due to RE_2_O_3_ incorporation filling the porous structure (Table 1) [27]. *I*–*V* measurements (Figure 3i) demonstrate a significant enhancement in the electrical conductivity of Yb_2_O_3_/ZnO, suggesting improved charge transport resulting from heterojunction formation.

**TABLE 1:** BET specific surface area, V_pore_, and D_pore_ of ZnO and RE_2_O_3_/ZnO nanostructures.

Sample	Surface area (m^2^ g^−1^)	V_pore_ (cm^3^ g^−1^)	D_pore_ (nm)
Yb_2_O_3_/ZnO	17.29	0.23	28.97
Y_2_O_3_/ZnO	18.08	0.11	22.70
Nd_2_O_3_/ZnO	16.51	0.21	49.09
ZnO	8.02	0.17	82.33

**FIGURE 4:**
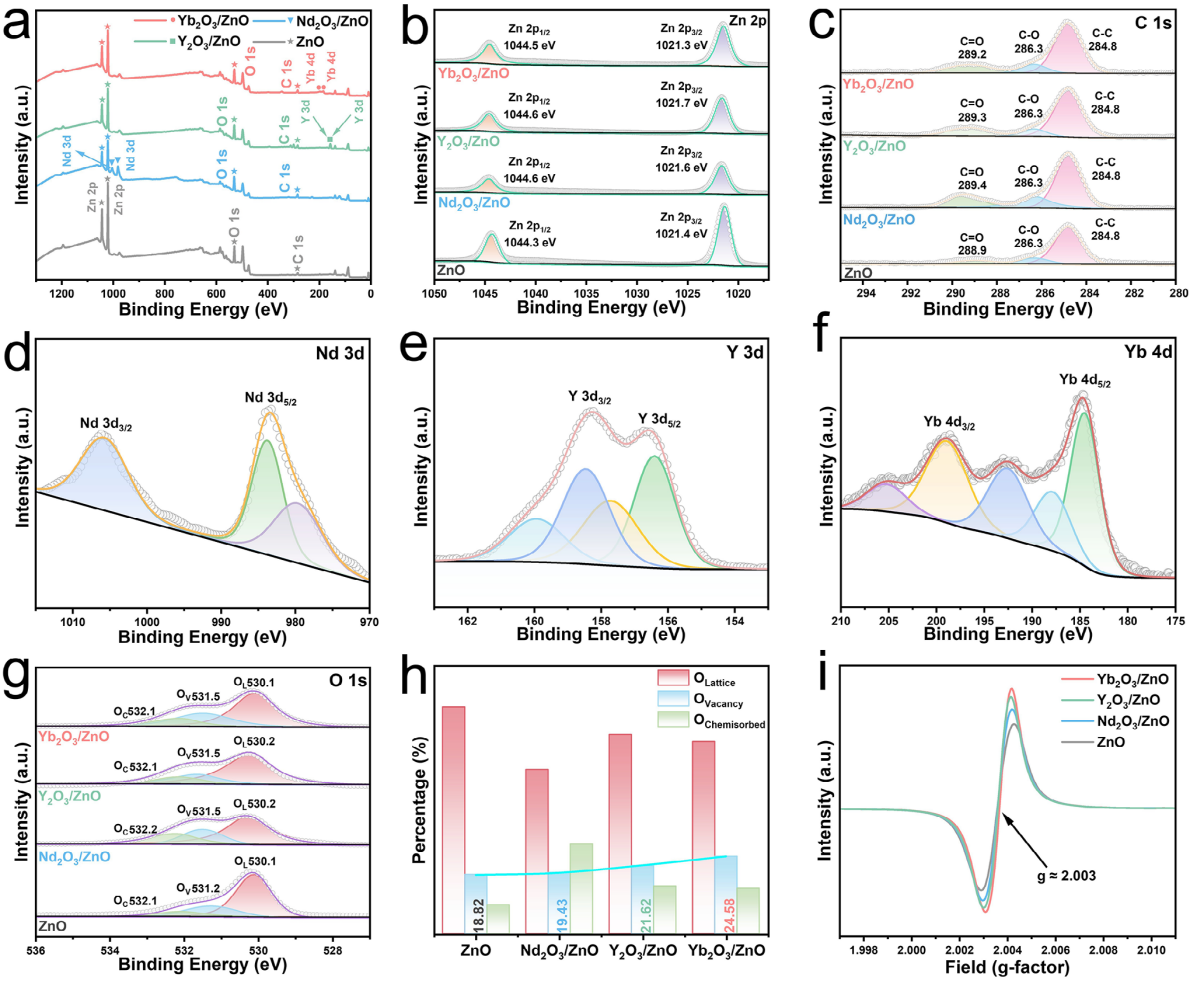
Surface chemical states and defect analysis of ZnO and RE_2_O_3_/ZnO (RE = Nd, Y, Yb) composites.(a) XPS survey spectra. (b–f) High‐resolution XPS spectra of (b) Zn 2p, (c) C 1s, (d) Nd 3d, (e) Y 3d, and (f) Yb 4d core levels. (g) Deconvoluted O 1s spectra. (h) Quantitative distribution of oxygen species. (i) EPR spectra.

The survey XPS spectrum (Figure 4a) confirms the presence of Zn, O, and C elements. High‐resolution spectra identify the characteristic peaks of Zn^2+^ in the Zn 2p region [28] (Figure 4b) and adventitious carbon in the C 1s region [29] (Figure 4c). The Nd 3d spectrum (Figure 4d) exhibits two prominent spin–orbit doublets, corresponding to the Nd 3d_5/2_ and Nd 3d_3/2_ states [30]. Deconvolution of the Nd 3d_5/2_ component reveals a peak at 980.2 eV, which is attributed to a charge transfer process from oxygen to the Nd 4f^3^orbital [31]. In the Y 3d spectrum of the Y_2_O_3_/ZnO sample (Figure 4e), a well‐separated doublet is observed, with peaks assigned to the Y 3d_5/2_ (156.5 eV) and Y 3d_3/2_ (158.5 eV) states [32]. Peaks at 159.9 and 157.7 eV are also present, corresponding to carbonate species formed from the reaction of Y_2_O_3_ with atmospheric CO_2_. For the Yb 4d spectrum (Figure 4f), the peaks located at 184.6 and 198.8 eV are assigned to Yb 4d_5/2_ and Yb 4d_3/2_, respectively [33]. Due to the presence of unpaired electrons in the 4f orbital of Yb^3+^, interactions between photoelectrons and valence electrons lead to energy loss. This oxidation state exhibits significant multiplet splitting resulting from Coulomb interactions, giving rise to characteristic peaks at 187.9, 192.7, and 205.2 eV [34].

The O 1s spectrum (Figure 4g) was fitted with three components, corresponding to lattice oxygen (O_L_), oxygen vacancies (O_V_), and chemisorbed oxygen (O_C_). Quantitative analysis (Figure 4h) demonstrates that the proportion of O_V_ in Yb_2_O_3_/ZnO reaches 24.58%, significantly higher than in the other samples. These abundant O_V_ serve as active sites for the adsorption and dissociation of atmospheric oxygen molecules, markedly increasing the concentration of reactive oxygen species on the material surface and thereby enhancing the response to the target gas [35].

The dominant role of O_V_ in Yb_2_O_3_/ZnO is further corroborated by the EPR spectroscopy (Figure 4i), which exhibits the strongest characteristic signal at g ≈ 2.003 among all samples. This pronounced signal is likely ascribed to the optimized charge transfer and chemical interactions from Yb_2_O_3_ to the ZnO matrix. The consistent findings from both XPS and EPR analyses clearly indicate that the Yb_2_O_3_/ZnO heterojunction, particularly after annealing at 500°C (Figure S8), facilitates the optimal formation of O_V_, thereby promoting the gas‐sensitive reaction.

### 2.2 Gas Sensing Characteristics of the Sample

**FIGURE 5:**
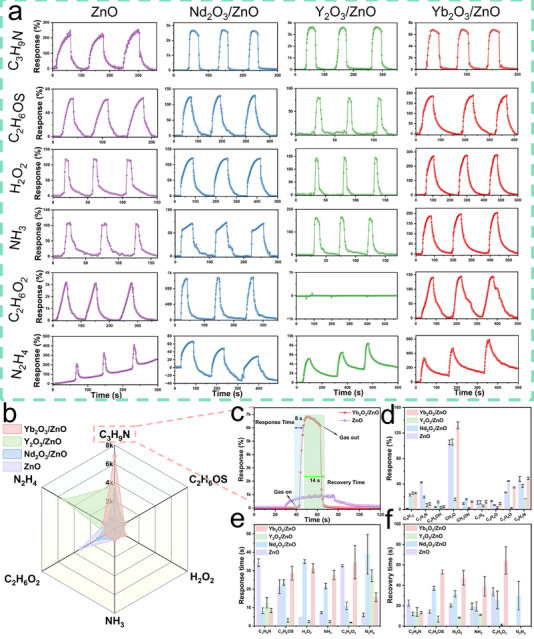
Gas‐sensing performance of ZnO and RE_2_O_3_/ZnO (RE = Nd, Y, Yb) sensors. (a) Dynamic response–recovery curves to 500 ppm of various target gases (TMA, C_2_H_6_OS, H_2_O_2_, NH_3_, C_2_H_6_O_2_, and N_2_H_4_). (b) Radar map illustrating selective response profiles. (c) Response and recovery characteristics to 500 ppm TMA for ZnO and Yb_2_O_3_/ZnO. (d) Comparison of sensor responses to 500 ppm of nine different analytes (C_9_H_12_, C_2_H_3_N, C_2_H_5_OH, CH_2_O, CH_3_OH, C_7_H_8_, C_3_H_6_O, C_7_H_6_O, and C_6_H_7_N). (e,f) Statistical analysis of the (e) response time and (f) recovery time across the sensor series.

The gas‐sensing performance of the ZnO and RE_2_O_3_/ZnO (RE = Nd, Y, Yb) sensorwereas systematically evaluated. As shown in Figure 5a, the dynamic response curves of the four sensors toward 500 ppm of various gases, including TMA, C_2_H_6_OS, H_2_O_2_, NH_3_, C_2_H_6_O_2_, and N_2_H_4_, were measured at RT. All sensors exhibited enhanced responses to reducing gases such as TMA and NH_3_, confirming their n‐type semiconductor behavior. Three consecutive response cycles further confirmed their good reversibility.

Among the samples, the RE_2_O_3_/ZnO sensors showed significantly enhanced responses to TMA compared to pure ZnO (Figure 5b) and to Yb_2_O_3_ sensors (Figure S9). Remarkably, the Yb_2_O_3_/ZnO sensor delivered the highest response value of 6.84k%, substantially exceeding the values of Y_2_O_3_/ZnO (3.63k%), Nd_2_O_3_/ZnO (2.68k%), and ZnO (229.3%). Furthermore, the Yb_2_O_3_/ZnO sensor demonstrated rapid response and recovery times of 8 s and 14 s, respectively (Figure 5c,d).

The selectivity of the four sensors was further examined against several common interfering gases (Figures S10–S12). Specificity was evaluated using the selectivity coefficient K, defined as K = S_TMA_ / S_other_, where S_TMA_ and S_other_ are the sensor responses to TMA and an interfering gas, respectively [36]. As summarized in Figure S13, all sensors displayed good selectivity. Among them, the Yb_2_O_3_/ZnO sensor exhibited the highest K values, each exceeding 16.9. Analysis of response and recovery times (Figure 5e,f) indicated that the Yb_2_O_3_/ZnO sensor also achieved the shortest sensing cycle for TMA detection. The generally longer recovery time relative to the response time suggests strong adsorption of TMA molecules on the sensing surface [37]. These results collectively demonstrate that the Yb_2_O_3_/ZnO sensor possesses excellent selectivity, high sensitivity, and favorable kinetics for TMA detection.

**FIGURE 6:**
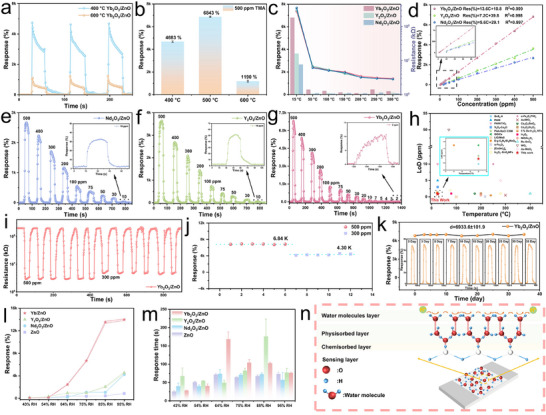
Optimized TMA Sensing Performance and Mechanism of RE_2_O_3_/ZnO Composites. (a) Dynamic response of Yb_2_O_3_/ZnO annealed at 400°C and 600°C toward 500 ppm TMA. (b) Corresponding response values. (c) Operating temperature‐dependent response of RE_2_O_3_/ZnO to 500 ppm TMA. (d) Concentration‐dependent response curves (2–500 ppm) with fitting. Dynamic response of (e) Nd_2_O_3_/ZnO, (f) Y_2_O_3_/ZnO, and (g) Yb_2_O_3_/ZnO to 2–500 ppm TMA. (h) Comparison of detection limits for Yb_2_O_3_/ZnO with reported sensors. (i) Repeatability test at 300 and 500 ppm TMA. (j) Statistical response values. (k) Long‐term stability at 500 ppm TMA over 30 days. Effect of relative humidity on (l) response magnitude and (m) response time. (n) Proposed humidity sensing mechanism.

The influence of annealing temperature on the Yb_2_O_3_/ZnO sensor was first investigated (Figure 6a,b). The sample annealed at 500°C exhibited the optimal response, outperforming those treated at 400°C and 600°C. Figure 6c (left axis) illustrates the variation in sensor response as a function of operating temperature. The response is highest at room temperature and gradually decreases as the temperature rises, while the baseline resistance in air (right axis) also shows a declining trend. This phenomenon can be attributed to the O_V_ in the samples, which form in‐gap defect states just below the conduction band. Many of these states are shallow donors, and their increased ionization at higher temperatures leads to a rise in charge carrier density [8].

The response range of the RE_2_O_3_/ZnO (RE = Nd, Y, Yb) sensors was evaluated by exposing them to TMA concentrations ranging from 2 to 500 ppm (Figure 6d). The dynamic response curves (Figure 6e–g) revealed a clear linear relationship at lower concentrations. The Yb_2_O_3_/ZnO sensor demonstrated the best linearity (Response = 13.6C + 10.8, R^2^ = 0.999), indicating strong potential for practical application. The limit of detection (LoD) for the Yb_2_O_3_/ZnO sensor at RT was calculated to be approximately 0.926 ppm using the IUPAC definition, $LoD=\frac{3S_{D}}{m}=(\frac{3}{m})\sqrt{\frac{\sum_{i=1}^{\;n}{(x_{i}-\bar{x})}^{2}}{n-1}}$, where m is the slope of the calibration curve and S_D_ is the standard deviation of the noise (Figure S14) [19]. This LoD is lower than the 5 ppm threshold set by the ACGIH, confirming the sensor's suitability for monitoring TMA exposure in compliance with safety standards [2]. A comparison with previously reported TMA sensors (Figure 6h; Table S1) highlights the high sensitivity and low detection limit of the Yb_2_O_3_/ZnO sensor at RT.

The stability of the Yb_2_O_3_/ZnO sensor was assessed via cyclic and long‐term tests. The sensor maintained excellent reproducibility over six consecutive cycles at both 500 and 300 ppm TMA (Figure 6i,j). Moreover, it demonstrated outstanding long‑term stability over 35 days, with a response value of 6933.6 ± 101.9% maintained during periodic testing (Figure 6k), confirming its high reliability.

Humidity tolerance is a key consideration for gas sensor applications. The responses of the four sensors under different RH levels (43%–95%) are shown in Figure 6l and Figure S15. Although the response of all sensors increases with humidity, the humidity response of Yb_2_O_3_/ZnO at 54% RH is 151%, indicating robust operation in moderately humid environments and suggesting relatively good humidity tolerance. However, in high‐humidity environments (>75% RH), its humidity response markedly increases, and the response time significantly lengthens (Figure 6m; Figure S16). The sensing mechanism under humid conditions is illustrated in Figure 6n. As RH rises, water molecules form a physisorbed layer via hydrogen bonding, facilitating proton conduction through a Grotthuss chain mechanism (*H_2_O + H_3_O^+^ → H_3_O^+^ + H_2_O*) [38], which substantially decreases sensor resistance. Surface modification strategies can be employed to mitigate this humidity interference [29].

Table 2 compares the TMA‐sensing performance of the as‐fabricated Yb_2_O_3_/ZnO sensor with other recently reported ZnO‐based sensors. Notably, the Yb_2_O_3_/ZnO sensor operates efficiently at RT, a significant advantage over most MOS sensors that require elevated temperatures. High operating temperatures not only accelerate sensor degradation but also restrict practical deployment. This limitation is particularly relevant for applications such as real‐time fish freshness monitoring, where RT operation is essential for both safety and feasibility [39]. The Yb_2_O_3_/ZnO sensor developed in this work thus exhibits distinctive benefits, including low‐energy operation, enhanced safety, improved stability, and broader applicability, underscoring its high practical utility. In summary, the Yb_2_O_3_/ZnO heterostructure demonstrates superior overall performance and energy efficiency. Furthermore, optimizing the annealing temperature is critical in maximizing its gas‐sensing response.

**TABLE 2:** Comparison of TMA sensing performance between Yb_2_O_3_/ZnO sensors and other ZnO based sensors reported in this work.

Sensing materials	Concentration (ppm)	Temperature (°C)	Response	Response and recovery time (s)	Refs.
CoZn15	50	100	∼85%^c^	19.4/15.5	[1]
ZnO‐MOF	20	140	270.1^a^	8/1205	[40]
Rh/ZnO	10	180	11.3^a^	93/110	[41]
ZnO‐3%Au	10	250	20.1^a^	12/127	[42]
Co_3_O_4_@ZnO	33	250	∼41^b^	3/2	[43]
ZnFe_2_O_4_/ZnO SAS30	100	240	31.5^a^	3.1/5.7	[5]
Pd‐doped ZnO nanorod arrays	5	300	5.5^a^	7/7	[6]
PdO‐decorated ZnO flower‐like structures	5	250	3.8^a^	14/12	[44]
ZnO	100	280	47^a^	19/8	[45]
Yb_2_O_3_/ZnO	500	25	6813%^c^	7/22	This work

^a^ Ra/Rg.

^b^ ΔR/Ra.

^c^ ΔR/Ra^*^ 100%.

### 2.3 Monitoring the Freshness of Fish

Accurate assessment of aquatic product freshness is critical for ensuring food safety. During spoilage, fish release biogenic amines such as NH_3_, TMA, and dimethylamine (DMA), whose concentration variations serve as key indicators of freshness [46].

**FIGURE 7:**
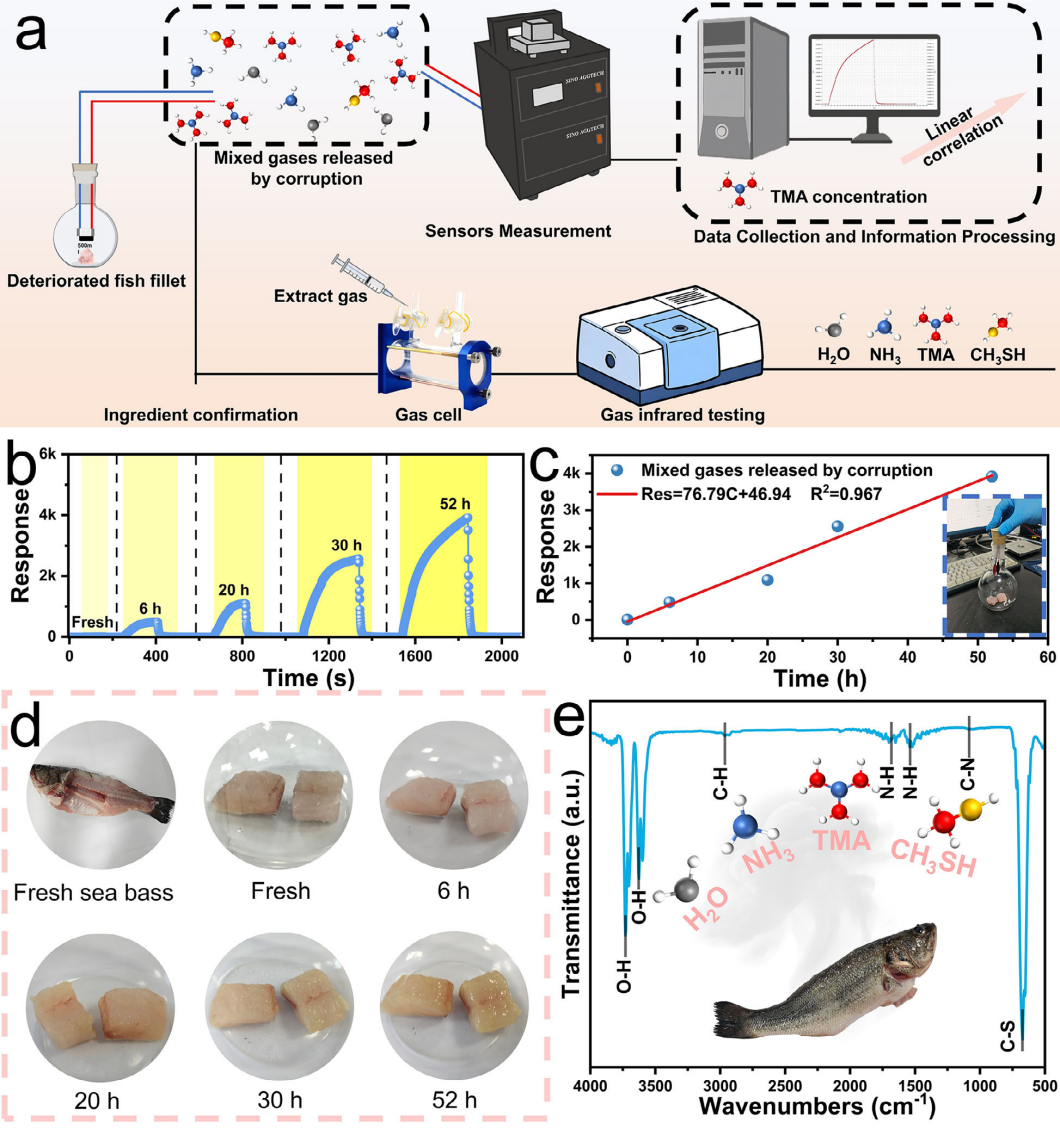
Real‐time Monitoring of Fish Freshness Using Yb_2_O_3_/ZnO Sensor. (a) Schematic of the fish freshness testing and product identification protocol. (b) Dynamic response and (c) corresponding fitted curves of the Yb_2_O_3_/ZnO sensor at RT toward volatile gases released from sea bass during spoilage over time. (d) Photographic images of sea bass at different storage time points. (e) FT‐IR spectra of the emitted spoilage gases.

To evaluate the practical potential of the Yb_2_O_3_/ZnO sensor, we applied it for real‐time monitoring of seafood freshness at RT. Sea bass, a high‐protein fish characterized by active microbial metabolism during spoilage, was selected. After rinsing with deionized water and drying, two 3 g samples were placed in 500 mL sealed flasks and stored at ambient temperature. During the monitoring process, the electrical signals were collected using the CGS‐MT comprehensive testing system. The sensor was connected to the system via leads equipped with rubber stoppers to ensure airtight integrity. For each measurement, the rubber stopper was temporarily removed, and the sensor leads were placed inside the bottle for testing. Data were recorded once the current response stabilized. Upon completion of the measurement, the bottle was immediately resealed and the leads were withdrawn, during which the resistance increased rapidly, completing one full monitoring cycle (Figure 7a).

Sensor responses were recorded at 0, 6, 20, 30, and 52 h, as illustrated in Figure 7b. The response increased significantly with storage time (Figure 7c), from 9.67 in the fresh state to 3918 after 52 h, indicating progressive TMA accumulation. A strong linear correlation (R^2^ = 0.967) was observed between the sensor response and storage duration, consistent with visual spoilage indicators such as flesh darkening, gelatinization, and liquid exudation (Figure 7d). FTIR analysis of volatile compounds (Figure 7e) further confirmed the presence of H_2_O, NH_3_, TMA, and CH_3_SH [47, 48, 49, 50, 51, 52].

Based on the selectivity test (Figure 5) and the sensor's response to CH_3_SH (405.7%, Figure S17), the fish spoilage response can be attributed primarily to TMA. Importantly, the responses under actual storage conditions consistently exceeded those in controlled humidity‐only environments (Figure S18), confirming that the signal originated mainly from spoilage gases rather than ambient humidity [16]. Sun et al. [53] reported that the hydrophilic nature of TMA and the presence of adsorbed water molecules on the sensor surface may promote TMA adsorption. These findings collectively affirm the strong practical applicability of the Yb_2_O_3_/ZnO sensor for real‐world food freshness monitoring.

### 2.4 Possible Gas Sensing Mechanisms

**FIGURE 8:**
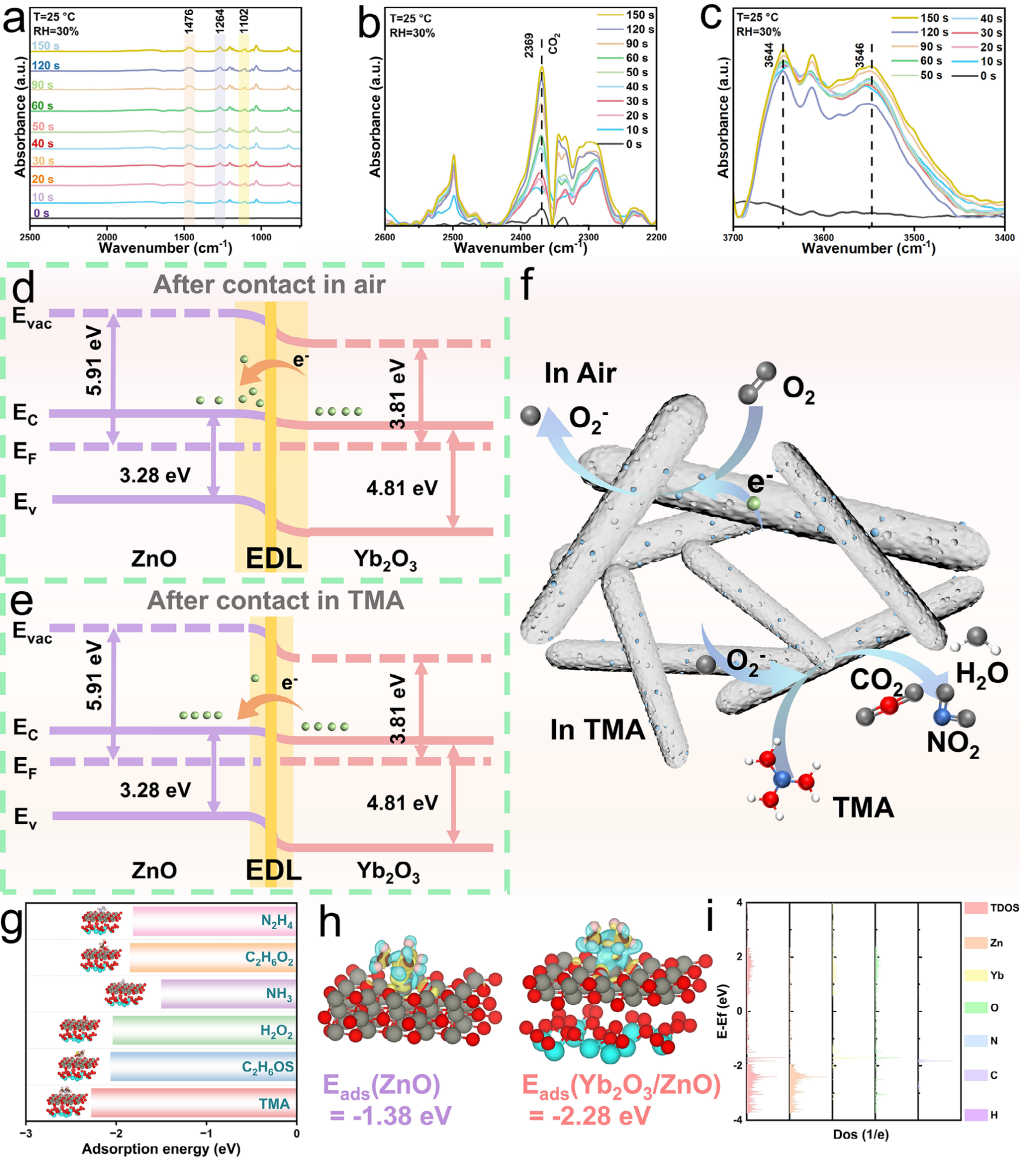
Reaction mechanism and electronic structure analysis of Yb_2_O_3_/ZnO for TMA sensing. (a–c) In situ DRIFTS spectra of Yb_2_O_3_/ZnO in TMA atmosphere at RT. (d,e) Band structure diagrams of Yb_2_O_3_/ZnO in (d) air and (e) TMA atmosphere. (f) Reaction mechanism schematic. (g) Adsorption energies of Yb_2_O_3_/ZnO toward TMA and five other highly responsive gases. (h) CDD diagram between ZnO and Yb_2_O_3_/ZnO. (i) DOS of TMA at the adsorption interface.

In situ diffuse reflectance infrared Fourier transform (DRIFT) spectroscopy further confirms the adsorption of TMA on Yb_2_O_3_/ZnO (Figure 8a–c). The broad peaks at 1476 and 1264 cm^−1^ correspond to the N═O stretching vibration of monodentate NO_2_^−^ and N─O stretching vibration [54]. respectively. The band at 1102 cm^−1^ is attributed to the O─O stretching mode of side‐bridged O_2_^−^ species [55]. The distinct sharp peak at 2369 cm^−1^ and broad peak in the range of 3644–3546 cm^−1^ likely originate from the presence of CO_2_ in the products [56] and O─H stretching vibrations [52], respectively. These observations confirm that NO_2_, CO_2_, and H_2_O are the primary reaction products.

To investigate the interfacial charge transfer in the Yb_2_O_3_/ZnO heterojunction, Mott–Schottky (M–S) analysis was performed (Figure S20). The positive slopes of the M–S plots confirm the n‐type nature of ZnO, Yb_2_O_3_, and the Yb_2_O_3_/ZnO heterojunction [57]. The measured flat‐band potentials (vs. SCE) were 0.096 V for ZnO, −0.424 V for Yb_2_O_3_, and −0.176 V for the Yb_2_O_3_/ZnO heterojunction. This intermediate flat‐band potential of the heterojunction indicates Fermi‐level alignment and the formation of a unified interfacial electronic structure. The ∼0.52 V difference in flat‐band potential between ZnO and Yb_2_O_3_ drives electron transfer from Yb_2_O_3_ to ZnO, creating a built‐in electric field (from Yb_2_O_3_ to ZnO) that is a key factor in enhancing the gas‐sensing performance.

It is well established that the thickness of the charge depletion layer (L) and particle size influence the gas‐sensing performance of semiconductors. Building on the above discussions, the underlying gas‐sensing mechanism is explained by the variation in the thickness of the semiconductor depletion layer.

The thickness of the charge depletion layer (L) is related to the surface density of adsorbed oxygen ions (N_t_) and the carrier concentration (N_d_) as described by Equation (1) [29]:

(1) $$L\;\propto \sqrt{\frac{N_{t}^{2}}{N_{d}^{2}}}=\frac{N_{t}}{N_{d}}$$

The measured E_g_ of Yb_2_O_3_ is 4.81 eV (Figure S21), and the φ values of ZnO and Yb_2_O_3_ are 5.91 and 3.81 eV, respectively (Figure S22); their band alignment is shown in Figure S23. Due to the formation of an n‐n heterojunction between Yb_2_O_3_ and ZnO, electrons transfer from Yb_2_O_3_ to ZnO, resulting in the formation of a potential barrier (qV1) at the interface (Figure 8d) [28]. This heterojunction barrier restricts current transport, where the resistance is determined by the height and width of the barrier; resistance is exponentially proportional to the effective barrier height (Equation 2) [58]:

(2) $$R=R_{0}\exp(\frac{qV}{kT})$$

When Yb_2_O_3_/ZnO is exposed to air, O_2_ molecules in the air capture electrons from the conduction band and adsorb onto the surface of the sensing material, reducing the carrier concentration, forming adsorbed surface oxygen ions, and thickening the electron depletion layer. At the Yb_2_O_3_/ZnO interface, the barrier height increases to q(V1 + V2) and the barrier width also expands [59], with the specific reaction Equation (3) and (4) [9]:

(3) $$O_{2}\left(\mathrm{gas}\right)\to O_{2}\left(\mathrm{ads}\right)$$

(4) $$\;O_{2}\left(\mathrm{ads}\right)+e^{-}\to O_{2}^{-}\left(\mathrm{ads}\right)$$

When the sensor is exposed to TMA, TMA molecules with low adsorption energy are adsorbed onto the surface and react with oxygen ions, producing NO_2_, CO_2_, and H_2_O while releasing electrons. These released electrons return to the conduction band of the gas‐sensing material, increasing the carrier concentration, narrowing the electron depletion layer, and thus reducing the material's resistance (Figure 8e,f; Figure S24), as described by Equation (5) [60]:

(5) $$4C_{3}H_{9}N+25O_{2}^{-}\to \;4NO_{2}+12CO_{2}+18H_{2}O+25e^{-}$$

At this point, the surface energy bands of Yb_2_O_3_ and ZnO bend, and the barrier height decreases to q(V1 + V2 – V3), enhancing the material's sensitivity and enabling TMA sensing at RT.

To further clarify the adsorption and desorption processes of TMA, Kelvin Probe Force Microscopy (KPFM) was employed to characterize the surface Contact Potential Difference (CPD). In KPFM, the CPD originating from the work function difference between the probe and the sample can be determined by detecting the resultant electrostatic force [61]. A voltage was applied to the probe, and the work function of the material (φ_*m*_) can be calculated using the following Equation (6) [62]:

(6) $$CPD=\frac{\varphi_{m}-\varphi_{tip}}{e}$$

where φ_*tip*_ is the work function of the probe and *e* is the elementary charge.

After exposure to TMA, the CPD of ZnO decreased from 290 to 263 mV (Figure S25a,b), while the CPD of Yb_2_O_3_/ZnO also decreased from 261 mV to 221 mV (Figure S25c,d). The decrease in CPD indicates a concurrent decrease in φ_*m*_​, which means the Fermi level of the sensing materials shifts toward the conduction band. This directly demonstrates that TMA, as a Lewis basic reducing gas, can transfer electrons to n‐type semiconductor materials, thereby regulating the charge accumulation process on the material surface [63].

Meanwhile, Figure S25e shows that the CPD variation of Yb_2_O_3_/ZnO (40 mV) is significantly larger than that of ZnO (27 mV), which indicates that the φ_*m*_ of Yb_2_O_3_/ZnO is lower than that of ZnO, consistent with the potential shift trend shown in Figure S25f,g. Therefore, the larger CPD variation of Yb_2_O_3_/ZnO confirms that the presence of the heterojunction results in higher interface charge separation efficiency. By strengthening the initial electron depletion layer and driving the directional separation of electrons generated from gas reactions, the weak surface chemical signal is amplified, thus achieving high‐sensitivity sensing of TMA at RT.

Meanwhile, the Bond strengths (and Ionization energies) of gases with high responses, such as TMA (C─N), C_2_H_6_OS (S = O), H_2_O_2_ (O─O), NH_3_ (N─H), C_2_H_6_O_2_ (C─C), and N_2_H_4_ (N─N), are 307 (7.82), 522, 142 (10.62), 386, 346, and 167 (8.1) kJ mol^−1^ (eV), respectively [64, 65]. It is evident that the C─N bond in TMA has relatively low bond strength and ionization energy, making its chemical bonds more easily broken and more reactive with adsorbed oxygen on the material surface. This enhances the selective detection of TMA by Yb_2_O_3_ and ZnO (Table S2) [9].

In this study, RE_2_O_3_/ZnO (RE = Nd, Y, Yb) sensors, particularly the Yb_2_O_3_/ZnO sensor, exhibit higher sensitivity in TMA detection for the following reasons:

- Morphologically, Yb_2_O_3_/ZnO retains a significantly higher number of surface pores compared to Y_2_O_3_/ZnO and Nd_2_O_3_/ZnO. This can possibly be attributed to the unique inhibitory effect of Yb^3+^ on ZnO grain growth, which is governed by a grain boundary pinning mechanism. The smaller ionic radius of Yb^3+^ (0.99 Å) promotes its segregation at ZnO grain boundaries, forming effective pinning sites that suppress atomic diffusion and grain agglomeration during annealing [66]. In contrast, the larger ionic radii of Nd^3+^ (1.12 Å) and Y^3+^ (1.02 Å) [67] limit this effective pinning capability, resulting in a less constrained sintering process that ultimately leads to pore collapse and material densification. The preserved porous structure in Yb_2_O_3_/ZnO facilitates the diffusion and adsorption of gas molecules within the material [19].
- XPS and EPR analyses confirm extensive lattice distortion at the Yb_2_O_3_/ZnO interface, generating a high density of O_V_. These O_V_ act as active adsorption sites, promoting the conversion of oxygen molecules to adsorbed O_2_^−^ and increasing L [68], thereby enhancing the adsorption capacity for TMA molecules as further supported by Tauc results. The excellent gas sensing performance of Yb_2_O_3_/ZnO can thus be attributed to the synergistic effects of O_V_ enrichment, interfacial charge transfer, and defect mediated conduction. This heterojunction not only optimizes the surface chemisorption of oxygen species but also facilitates efficient charge separation.
- The ionic radius of the trivalent RE ion Yb^3+^ is smaller than that of Y^3+^ and Nd^3+^, resulting in higher charge density and stronger ability to attract electron pairs. The strong Lewis acidity of Yb^3+^ makes it more effective at polarizing O_2_^−^, reducing the formation energy of O_V_ [69]; this also helps attract more Lewis basic gases (e.g., TMA) in chemical sensing, improving the material's adsorption performance. The 4f^13^ configuration of Yb^3+^ endows its electron cloud with high polarizability [70], further strengthening its interaction with O_2_^−^. In contrast, Y^3+^ (4f^0^) and Nd^3+^ (4f^4^) exhibit weaker Lewis acidity due to their larger radii and lower charge density, leading to insufficient polarization of O_2_^−^ and weaker O_V_ regulation effects. This ultimately results in significantly superior gas‐sensing performance of Yb_2_O_3_/ZnO compared to Y_2_O_3_/ZnO and Nd_2_O_3_/ZnO.
- Simultaneously, the gas‐sensing performance of Yb_2_O_3_/ZnO is analyzed using the thermionic emission theory of Schottky barriers (Equation (7)) [71]:
(7) $$J\propto \;\exp(-\frac{q\varphi_{B}}{kT})$$

In the formation of heterojunctions, Y_2_O_3_/ZnO (3.91 eV) and Nd_2_O_3_/ZnO (2.71 eV) (Figure S26) [72, 73, 74] with higher barrier heights (φ_B_) exhibit lower current density (J) across the interface, drastically reducing the efficiency of electron transport over the barrier. Thus, the competition between the optimized gas‐sensing performance from the thickened L induced by high φ_B_ and the reduced J contributes to the differences in gas‐sensing performance among various RE_2_O_3_/ZnO samples, whereas Yb_2_O_3_/ZnO (2.1 eV) achieves an optimal balance in φ_B_.

The formation of n‐n heterojunctions on nanocomposites is conducive to carrier modulation and facilitates the enhancement of gas‐sensing performance. Therefore, this study further employs DFT calculations to investigate the effect of the presence of heterojunctions in the gas‐sensing properties of the material.

The adsorption energy (*E*_ads._) was calculated using Equation (8) [10]:

(8) $$E_{\mathrm{ads}.}=E_{\mathrm{total}}-E_{\mathrm{substrate}}-E_{\mathrm{gas}}$$

In the equation, *E*_total_, *E*_substrate_, and *E*_gas_ denote the total energy of gas molecules adsorbed on the substrate, the energy of the substrate, and the energy of the original gas molecules, respectively.

CDD (△*ρ*) is obtained using Equation (9) [58]:

(9) $$\Delta \rho =\rho_{\mathrm{total}}\;-\;\rho_{\mathrm{substrate}}\;-\;\rho_{\mathrm{gas}}$$

In the equation, *ρ*_total_, *ρ*_substrate_, and *ρ*_gas_ denote the total charge density of the gas‐adsorbed substrate, the total charge density of the substrate, and the total charge density of the isolated gas molecules, respectively.

Herein, we calculated the adsorption energies of TMA molecules on ZnO and Yb_2_O_3_/ZnO. The results showed that the adsorption energy of TMA on Yb_2_O_3_/ZnO (−2.28 eV) was significantly higher than that on ZnO (−1.38 eV). For a gas‐sensing material, a higher adsorption energy toward the target gas implies that more target gas molecules can be adsorbed, which in turn generates a more pronounced sensing signal and enhances sensitivity. Analysis of the adsorption energy data and optimized adsorption configurations of the surface layer for Yb_2_O_3_/ZnO (Figure 8g) revealed significant interactions between Yb_2_O_3_/ZnO and TMA, as well as five other high‐response gas molecules (C_2_H_6_OS, H_2_O_2_, NH_3_, C_2_H_6_O_2_, and N_2_H_4_). Among these gases, TMA exhibited the most prominent adsorption behavior, with the absolute value of its adsorption energy substantially exceeding that of the other tested gases. In terms of adsorption energy magnitudes, a value with an absolute value less than 0.8 eV is generally categorized as physical adsorption, while values above this threshold are defined as chemical adsorption [75]. Simulation results demonstrated that all six gas molecules bound to the sensor material surface via chemical adsorption, confirming the chemical adsorption nature of the Yb_2_O_3_/ZnO system. Thus, the computational results indicate that the incorporation of Yb_2_O_3_ facilitates an increase in the adsorption energy of ZnO toward TMA, thereby improving its gas‐sensing performance.

In the comparative calculations of CDD for ZnO and Yb_2_O_3_/ZnO (Figure 8h), the isosurface threshold was set to 0.001 e Å^−3^, where yellow denotes charge accumulation and blue denotes charge depletion. The results showed that the reductive TMA molecule was surrounded by a charge depletion region, exhibiting an electron‐donating property. The interaction between TMA and the adsorption sites of Yb_2_O_3_/ZnO induced electron transfer; notably, the formation of a layered structure with charge accumulation enhanced the interaction between the gas molecule and the material. This observation indicates that the incorporation of Yb_2_O_3_ further strengthens the material's ability to accept and donate electrons toward TMA.

PDOS analysis reveals (Figure 8i) that after TMA adsorption, new electronic states emerge near the Fermi level; these states originate primarily from the O and Zn states of the Yb_2_O_3_/ZnO heterostructure, while the states associated with TMA are mostly localized below the valence band edge. This indicates that electrons from TMA are transferred to the oxide host, leading to an increase in carrier density. Meanwhile, the enhanced DOS reduces the energy barrier for electron transition, thereby increasing electrical conductivity and ultimately enabling a high response value through changes in resistance. This provides a mechanistic basis for understanding the material's gas‐sensing response.

## 3 Conclusion

In this work, four sets of gas sensors, which include pure ZnO and rare‐earth (RE) oxide modified ZnO heterojunctions (RE_2_O_3_/ZnO, RE = Nd, Y, Yb), were successfully fabricated via the coprecipitation method, and among these, the Yb_2_O_3_/ZnO sensor achieved highly sensitive and selective detection of TMA at RT, exhibiting a TMA response of 6.84 K%, a theoretical LoD of 0.926 ppm, and excellent long‐term stability. Furthermore, this sensor showed a favorable linear correlation with the freshness assessment of sea bass, showcasing significant potential for practical applications. This outstanding performance is primarily attributed to the unique porous structure of Yb_2_O_3_/ZnO, its heterojunction effect, O_V_ modulation, and the strong catalytic role of Yb_2_O_3_ in regulating O_V_, while DFT calculations were employed to investigate the influence of Yb_2_O_3_ incorporation on sensor performance, elucidating the origin of the excellent selectivity and providing theoretical support for understanding the gas adsorption process and charge distribution. Thus, this study holds significant theoretical and practical implications for the development of RT gas sensors based on RE_2_O_3_/ZnO composites.

## 4 Experimental Section

Supporting Information (SI) provides detailed information on specific materials, material characterization equipment, humidity detection details (Figure S27), the fabrication process of gas‐sensing sensors via conventional coating techniques and the DFT calculation methods employed in this study.

### 4.1 Synthesis of ZnO and RE_2_O_3_/ZnO (RE = Nd, Y, Yb)

Synthesis of ZnO: 0.68 g of zinc chloride (ZnCl_2_), 0.5 g of polyvinylpyrrolidone (PVP K30), and 0.8 g of sodium hydroxide (NaOH) were sequentially added to 25 mL of deionized (DI) water. After magnetic stirring for 3 h, a white turbid solution was obtained, which was then allowed to stand at RT for 18 h. Following precipitation, the supernatant was removed using a dropper, and the precipitate was transferred to a centrifuge tube. The precipitate was alternately washed with DI water and absolute ethanol three times, centrifuged at 10 000 rpm to remove impurities, and then dried in an oven at 100°C for 10 h. Finally, calcination was performed in a tube furnace at 500°C for 4 h with a heating rate of 5°C min^−1^, yielding white ZnO powder. The overall synthesis procedure was identical to that described above (Figure 1), except that 0.285 g of neodymium nitrate hexahydrate (Nd(NO_3_)_3_·6H_2_O), 0.249 g of yttrium nitrate hexahydrate (Y(NO_3_)_3_·6H_2_O), and 0.292 g of ytterbium nitrate pentahydrate (Yb(NO_3_)_3_·5H_2_O) were added separately prior to the initial magnetic stirring. These samples were designated as Yb_2_O_3_/ZnO, Y_2_O_3_/ZnO, and Nd_2_O_3_/ZnO, respectively. Likewise, Yb_2_O_3_ powder was obtained.

### 4.2 Fabrication of ZnO and RE_2_O_3_/ZnO (RE = Nd, Y, Yb) Sensors and Gas‐Sensing Measurements

The gas‐sensing performance of the sensors was evaluated using a comprehensive photoelectric testing platform (CGS‐MT, Beijing, China) at temperatures ranging from RT to 300°C under a relative humidity (RH) of 30 ± 5%. The CGS‐MT unit employed for testing is an integrated system whose standard configuration includes a built in temperature controlled 1000 mL gas generation chamber. After connecting the sensor to the system, the current change induced by the target gas was recorded in I–T mode under a bias of 4 V. For n‐type semiconductors, the sensor response was defined as “ $Response(\%)=\frac{\Delta I}{I_{\mathrm{air}}}=\frac{I_{\mathrm{gas}}\;-\;I_{\mathrm{air}}}{I_{\mathrm{air}}}\;\times 100\%$” or “$Response=\frac{\Delta I}{I_{\mathrm{air}}}=\frac{I_{\mathrm{gas}}\;-\;I_{\mathrm{air}}}{I_{\mathrm{air}}}$”, where I_gas_ and I_air_ denote the current in the target gas and clean air, respectively. The response time and recovery time were defined as the time required to reach 90% of the stable response value and to recover to 10% of the stable value, respectively. For high‐temperature tests, the CGS‐MT system was pre‐set to the target temperature, and measurements were performed only after the baseline had stabilized.

The gases measured in the experiment were prepared using thermal evaporation. A microsyringe was used to inject the target liquid into a 1000 mL cylinder. Different evaporation temperatures were set according to the boiling points of the target gases. Subsequently, the liquid injected into the cylinder evaporated and was purged by the cylinder's built‐in fan. Probes connected to the two ends of the interdigitated electrodes coated with sensitive material formed a closed circuit, enabling the evaluation of the material's gas‐sensing performance. The thermal evaporation formula for VOCs is as shown in Equation (10) [37]:

(10) $$Q=\frac{V\cdot C\cdot M}{22.4\cdot \omega \cdot \rho }\;\times 10^{-9}\times \frac{273+T_{R}}{273+T_{C}}$$

where *Q* (mL) is the volume of the target liquid to be taken with the microsyringe; *V* (mL) is the volume of the gas cylinder; *C* (ppm) is the concentration of the test gas; M (g mol^−1^) is the molecular weight of the liquid; *ω* (%) is the mass percentage of the target liquid; *ρ* (g mL^−1^) is the density of the target liquid; and *T*_R_ (°C) and *T*_C_ (°C) are the ambient temperature and the temperature inside the gas cylinder during testing, respectively (The specific parameters required for testing gas concentration are presented in Table S3). The sensor fabrication and testing processes are illustrated in Figure 1.

### 4.3 Statistical Analysis

All data with error bars were obtained from at least three independent samples and are presented as mean ± standard deviation (SD). The data were plotted with Origin software, and statistical analyses were performed with Microsoft Excel.

## Funding

This research was funded by Tianshan Talent Training Project‐Xinjiang Science and Technology Innovation Team Program (2023TSYCTD0012), Xinjiang Natural Science Fund for Distinguished Young Scholars (2022D01E37), Key programs of Xinjiang Natural Science Foundation (2024LQ01001‐3, 2022B02051), Tianshan Innovation Team Program of Xinjiang Uygur Autonomous Region (2023D14001), Xinjiang Tianshan Talent Project (2024TSYCCX0007), and Xinjiang University Innovation Project for PhD candidate (XJU2024BS047).

## Conflicts of Interest

The authors declare no conflicts of interest.

## Supporting information




**Supporting File**: advs74025‐sup‐0001‐SuppMat.docx.

## Data Availability

The data that support the findings of this study are available from the corresponding author upon reasonable request.
