# Supplementary material for: Interfacial Engineering in Rare‐Earth Oxide/ZnO Heterojunctions for High‐Performance Trimethylamine Sensing and Fish Freshness Monitoring
Source: Adv Sci (Weinh). 2026 Jan 22;13(19):e22490. doi: 10.1002/advs.202522490 (PMC13045216; doi:10.1002/advs.202522490)
Supplement: Supplementary file 1 — Supporting File: advs74025‐sup‐0001‐SuppMat.docx. [file ADVS-13-e22490-s001.docx]

Supporting Information

**Interfacial Engineering in Rare-Earth Oxide/ZnO Heterojunctions for High-Performance Trimethylamine Sensing and Fish Freshness Monitoring**

Junxi Cheng ^#,a,c^, Chang Liu ^#,a,c^, Miaomiao Liu ^b^, Qihua Sun ^b^, Jun Sun ^b,*,1^, Zhaofeng Wu ^a,b,c,*,1^

*^a^ School of Materials Science and Engineering, Xinjiang University, Urumqi, Xinjiang 830046, China*

*^b^ School of Physics Science and Technology, Xinjiang University, Urumqi, Xinjiang 830046, China*

*^c^ Xinjiang Engineering Research Center for Environmental Functional Materials, Xinjiang University, Urumqi, Xinjiang 830046, China*

^#^ These authors contributed equally to this work.

Corresponding author E-mail: sunjun@xju.edu.cn (J. Sun), wuzf@xju.edu.cn (Z. Wu)

**1. Experimental Section**

*1.1 Materials*

All chemical reagents used in the preparation of nanocomposites are analytical grade (AR) and do not require further purification. The reagents required for the synthesis of sensitive experimental materials: zinc chloride (ZnCl_2_), polyvinylpyrrolidone (PVP_K30_), and sodium hydroxide (NaOH) were purchased from Aladdin Chemical Reagent Co., Ltd. Yttrium nitrate pentahydrate (Yb(NO_3_)_3_·5H_2_O), Yttrium nitrate hexahydrate (Y(NO_3_)_3_·6H_2_O), and neodymium nitrate hexahydrate (Nd(NO_3_)_3_·6H_2_O) were purchased from Shanghai McLean Chemical Reagent Co., Ltd. Anhydrous ethanol was purchased from China National Pharmaceutical Group Chemical Reagent Co., Ltd. DMSO (C_2_H_6_OS), Hydrazine (N_2_H_4_), Ammonia (NH_3_), Aniline (C_6_H_7_N), Benzaldehyde (C_7_H_6_O), Acetone (C_3_H_6_O), Toluene (C_7_H_8_), Methanol (CH_3_OH), Formaldehyde (CH_2_O), Acetonitrile (CH_3_CN), 1,3,5-Trimethylbenzene (C_9_H_12_), Hydrochloric acid (HCl). Ethanol (C_2_H_6_O), Ethylenediamine (C_2_H_8_N_2_), Glycol ((CH_2_OH)_2_) was purchased from Sinopharm Chemical Reagent Co., Ltd. Purchase of Aladdin Chemical Reagent Co., Ltd. for Hydrogen peroxide (H_2_O_2_) and Trimethylamine (C_3_H_9_N).

*1.2 Materials characterization*

The crystal structure and crystallinity of the samples were analyzed using powder X-ray diffraction (XRD, Bruker D8 Advance, Germany) with a 2θ scanning range of 5°–90° using Cu-Kα radiation (λ = 1.5418 Å), allowing for the identification of phase compositions. Surface morphologies were observed via cold field emission scanning electron microscopy (SEM, JSM-6700F, Germany). Transmission electron microscopy (TEM, JEOL JEM-F200, Japan) was employed to characterize internal microstructures, while energy-dispersive X-ray spectroscopy (EDS) integrated with the TEM system provided insights into elemental distributions and synthetic dimensions. X-ray photoelectron spectroscopy (XPS, Kratos AXIS SUPRA, Japan) was utilized to determine chemical valence states and elemental compositions, with charge correction applied using the C1s peak at 284.8 eV to ensure spectral accuracy. Electron paramagnetic resonance (EPR, Bruker A300, Germany) was used to study the magnetic resonance behavior of unpaired electrons in paramagnetic species. Raman spectroscopy (LabRam HR Evolution, Japan) with a 532 nm laser excitation source was employed to analyze molecular structures and chemical bonding environments. Thermal degradation profiles were obtained via simultaneous thermogravimetric-differential thermal analysis (TGA, SDT650, USA). Ultraviolet-visible spectroscopy (UV-VIS, UV-3600 Plus, Japan) was used to measure absorption spectra for structural characterization. Fourier-transform infrared spectroscopy (FT-IR, BRUKER TENSOR II, Germany) was performed to identify functional groups. Finally, Brunauer-Emmett-Teller (BET) analysis (Micromeritics ASAP2460, USA) characterized specific surface areas and pore size distributions through N₂ adsorption-desorption isotherms.

*1.3* *Humidity detection*

In the humidity sensing test of sensors, saturated aqueous solutions of K_2_CO_3_, Mg(NO_3_)_2_, CuCl_2_, NaCl, KCl and KNO_3_ were used to obtain relative humidity environments of 43% RH, 54% RH, 64% RH, 75% RH, 85% RH, and 95% RH (Figure S27).[1, 2] Conduct humidity resistance tests on the sensors in different relative humidity environments mentioned above, and obtain real-time current response values after they stabilize. The definition of humidity response is as follows: $Response(\%)=\frac{\Delta I}{I_{h}}=\frac{I_{h}-I_{a}}{I_{a}}\times100\%$, where I_h_ is the current of the sensor at 43% RH, 54% RH, 64% RH, 75% RH, 85% RH, and 95% RH, and I_a_ is the current of the sensor at 30% RH.

*1.4 Fabrication of ZnO and RE_2_O_3_/ZnO (RE = Nd, Y, Yb) sensors*

To ensure uniform coating of the sample on the sensor surface, a portion of the sample was weighed and ground thoroughly with a defined amount of deionized water in an agate mortar (at a powder-to-water ratio of 4:1) to form a homogeneous paste. Subsequently, the paste was dropped onto the interdigitated Ag/Pd electrodes (with a mass ratio of 95:5 for Ag to Pd) on an Al_2_O_3_ ceramic substrate (13×7×0.635 mm), followed by air-drying at RT for 2 weeks.

*1.5 DFT Calculation Details*

DFT calculations were performed using the Vienna Ab initio Simulation Package (VASP) [3], employing the Projector-Augmented Wave (PAW) method [4]. Exchange-correlation interactions between electrons were treated within the spin-polarized generalized gradient approximation (GGA) with the Perdew-Burke-Ernzerhof (PBE) functional.[5] The supercell structure of the Yb_2_O_3_/ZnO heterojunction was generated using the VASPKIT code;[6] within this supercell, the lattice mismatch between Yb_2_O_3_ and ZnO was below 3.94%, and a 15 Å vacuum layer was introduced to eliminate interference between adjacent systems. For surface energy calculations, Brillouin zone sampling was conducted using a 2×2×1 k-point grid. To construct reconstructed models, the (110) surface of ZnO and the (100) surface of Yb_2_O_3_ were first built using 2×2 supercells composed of primitive cells; subsequently, an appropriate amount of zinc (Zn), ytterbium (Yb), and oxygen (O) atoms were inserted into interstitial sites, resulting in the generation of multiple structural models. The cutoff energy for the plane-wave basis set was set to 500 eV, and the convergence energy threshold for self-consistent calculations was 1×10^-5^ eV. During the optimization of equilibrium geometric structures, the maximum stress acting on each atom was constrained to not exceed 0.02 eV/Å. Dipole correction was considered for all systems.

**2. Results and discussions**

**
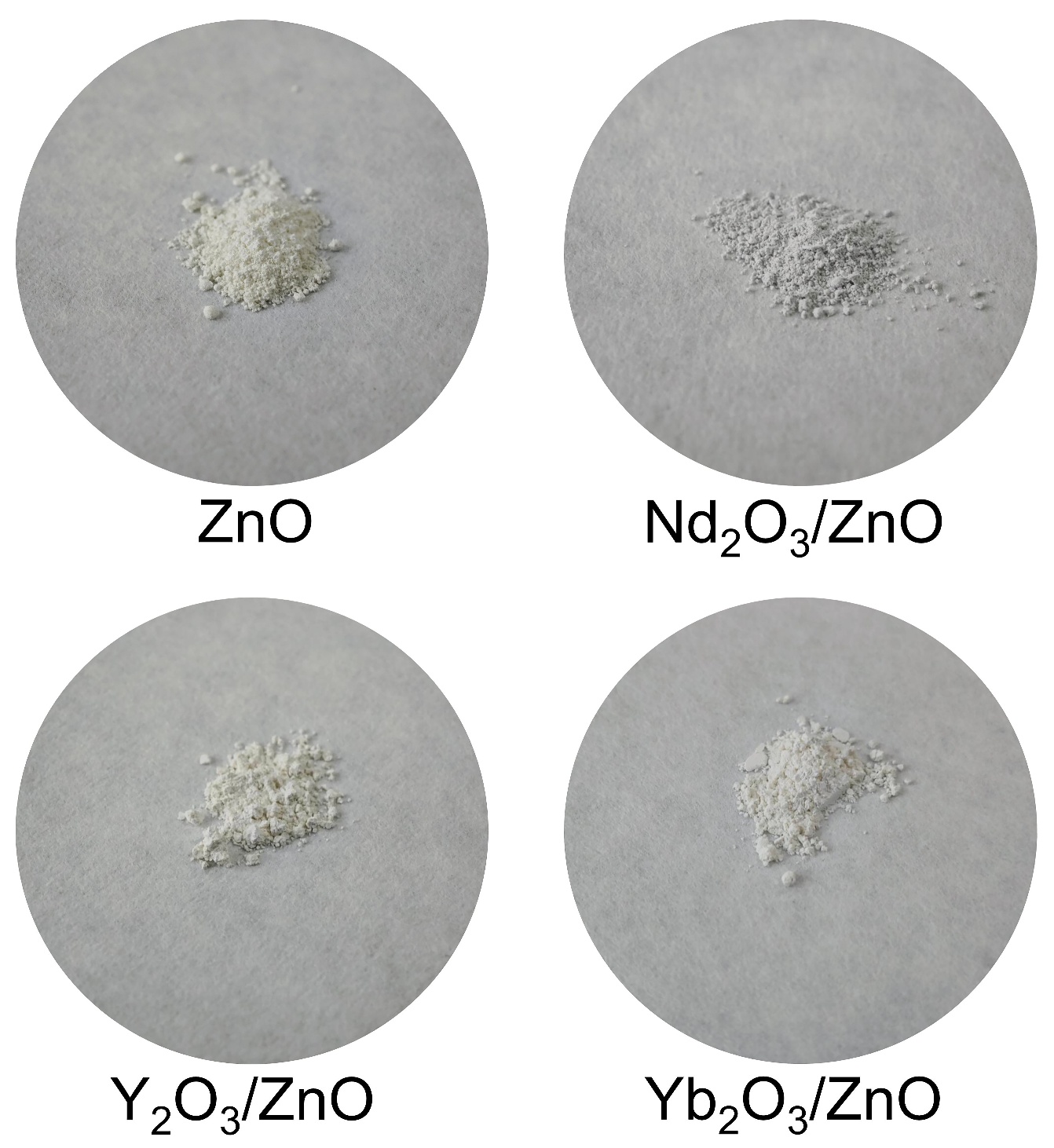
**

**Figure S1.** Appearance of ZnO and RE_2_O_3_/ZnO (RE=Nd, Y, Yb) samples.


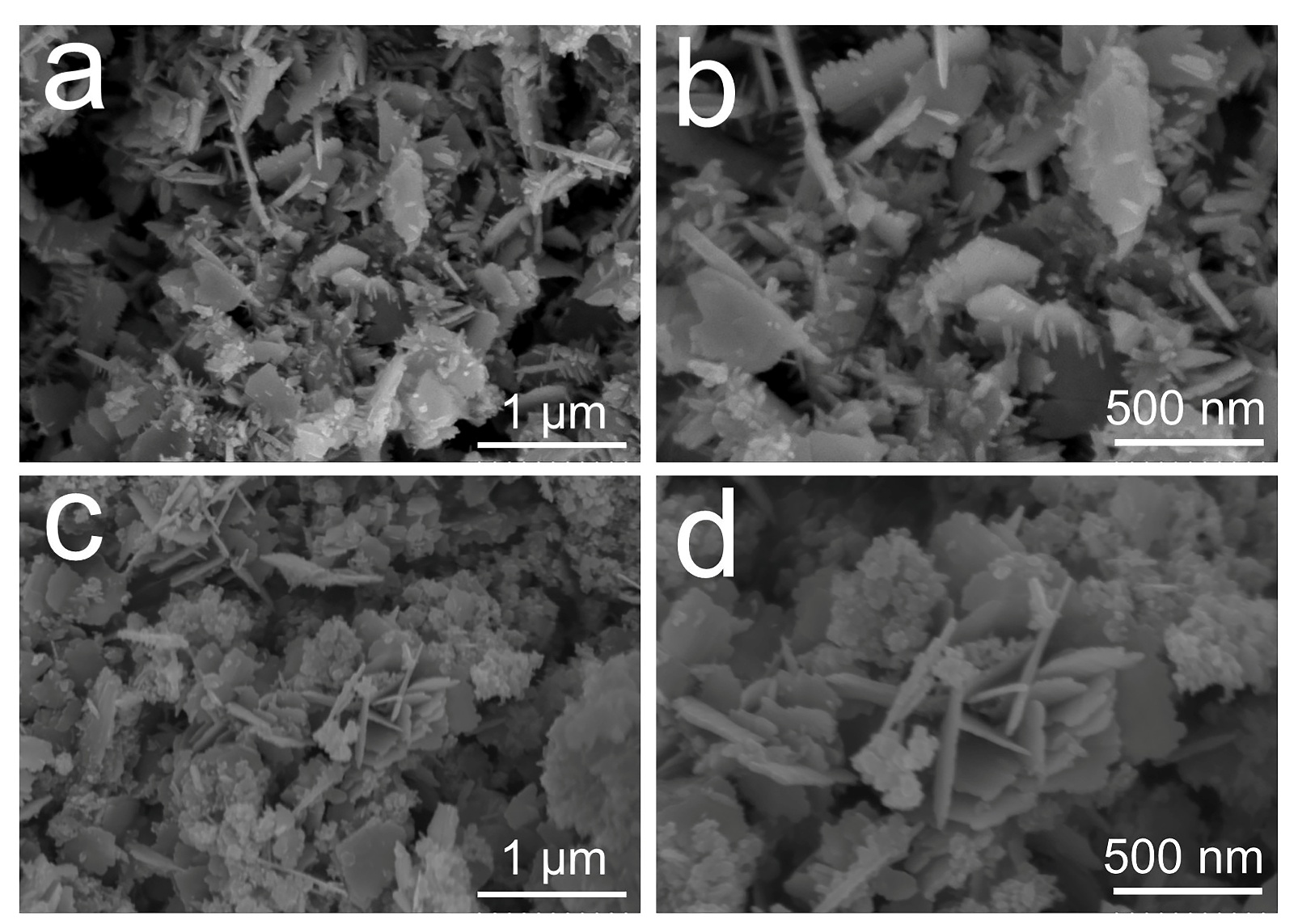


**Figure S2.** SEM images of a-b) Y_2_O_3_/ZnO before annealing and c-d) Nd_2_O_3_/ZnO before annealing.


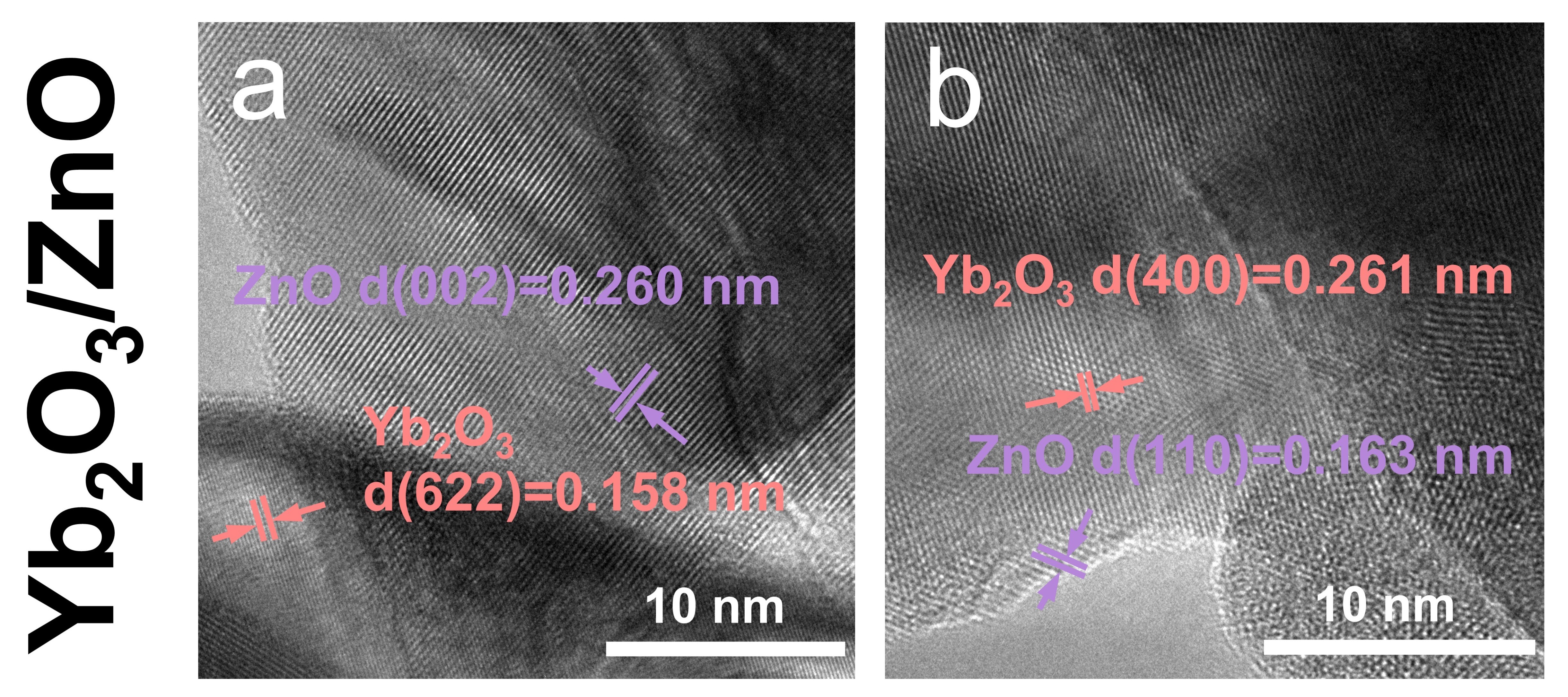


**Figure S3.** HRTEM image of Yb_2_O_3_/ZnO.





**Figure S4.** XRD pattern of Yb_2_O_3_.





**Figure S5.** DCS spectra of ZnO and RE_2_O_3_/ZnO (RE=Nd, Y, Yb) samples.





**Figure S6.** UV-Vis spectra of ZnO and RE_2_O_3_/ZnO (RE=Nd, Y, Yb) samples.





**Figure S7.** VBM spectra of ZnO and RE_2_O_3_/ZnO (RE=Nd, Y, Yb) samples.

Figure S8a presents the XPS survey spectra of Yb_2_O_3_/ZnO annealed at 400 ℃ and 600 ℃. For Zn 2p (Figure S8b), the peaks centered at 1021.3 eV and 1044.1 eV in the 400 ℃ sample are consistent well with the spectral features of the 500 ℃ sample. When the annealing temperature is increased to 600 ℃, the intensity of Zn 2p satellite peaks significantly enhances, indicating exacerbated lattice distortion. For C 1s (Figure S8c), the 600 ℃ sample exhibits increased intensity of surface carbon-containing functional groups such as C=O and C-O. This suggests that surface carbon species may undergo re-oxidation of residual carbon under the synergistic effect of high temperature, defect enrichment, and rare-earth catalysis. Yb 4d (Figure S8d) also shows distinct differences, with changes in both the position and intensity of main peaks and satellite peaks. This could arise from the diffusion of Yb^3+^ into the ZnO lattice induced by high temperature, which triggers the reconstruction of interfacial Yb-O-Zn bonds. For O1s (Figure S8e, f), the 400 ℃ sample exhibits high lattice regularity with few defects and surface contaminants, retaining the characteristics of intrinsic lattice oxygen. The proportion of O_V_ is lower than that of the 500 ℃ sample, which limits its gas-sensing performance. When the temperature is raised to 600 ℃, the distribution of oxygen species undergoes a drastic transformation: O_L_ decreases to 21.73%, O_C_ increases to 58.41%, and O_V_ drops to 19.86%. High temperature disrupts O_L_, promoting the migration of bulk O_L_ to the surface to form stable adsorbed species. Meanwhile, O_C_ fills part of the O_V_, reducing its proportion and similarly inhibiting gas-sensing performance.


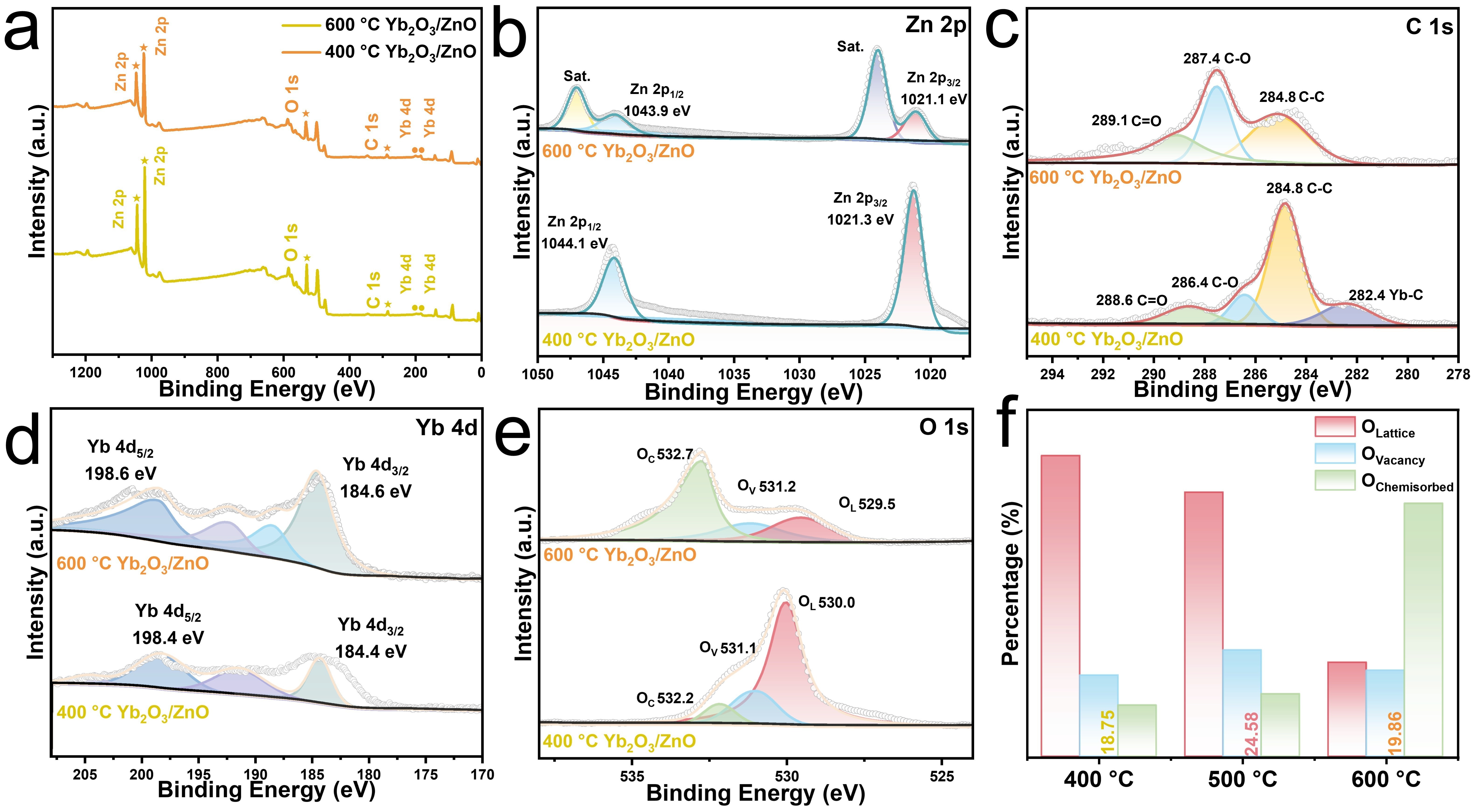


**Figure S8.** XPS full spectrum and detailed spectrum of Yb_2_O_3_/ZnO samples annealed at 400 ℃ and 600 ℃, respectively: a) XPS full spectrum, b) Zn 2p, c) C 1s, d) Yb 4d, e) O 1s. f) The proportion of O_L_, O_V_, and O_C_ in the sample.

The dynamic response recovery curves in Figure S9a show that pure Yb_2_O_3_ exhibits moderate responses to 15 volatile organic compounds (VOCs) at 500 ppm. Its response to 500 ppm TMA is 113.7%, which is significantly lower than that of the Yb_2_O_3_/ZnO heterostructure (6840%) and pure ZnO (229.3%). The selectivity radar chart in Figure S9b highlights the selective response profile of Yb_2_O_3_, indicating notably higher responses to gases such as C_7_H_6_O (3143%) and C_6_H_7_N (1046%), reflecting its intrinsic selectivity. Figure S9c provides the response and recovery times of Yb_2_O_3_ to 500 ppm TMA, confirming its functionality as a room temperature sensor. These results demonstrate that while pure Yb_2_O_3_ possesses detectable gas sensing activity, its performance is substantially enhanced when combined with ZnO to form a heterojunction.


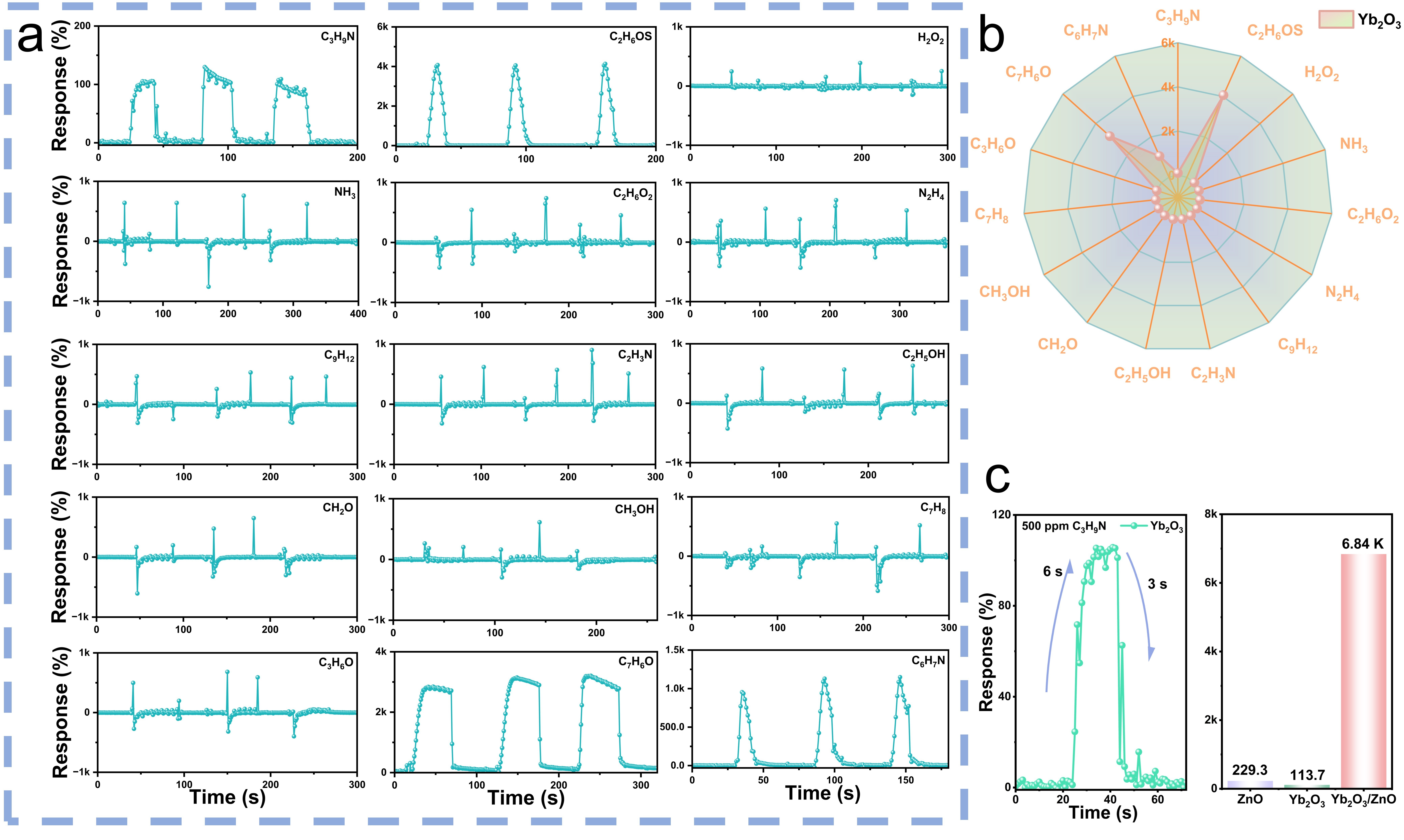


**Figure S9.** Gas-sensing performance of the Yb_2_O_3_ sensor. a) Dynamic response-recovery curves towards 15 types of VOCs at a concentration of 500 ppm. b) Radar chart illustrating the selective response profiles. c) Response and recovery time of the Yb_2_O_3_ sensor towards 500 ppm TMA, as well as the response values of ZnO, Yb_2_O_3_, and Yb_2_O_3_/ZnO sensors towards 500 ppm TMA.


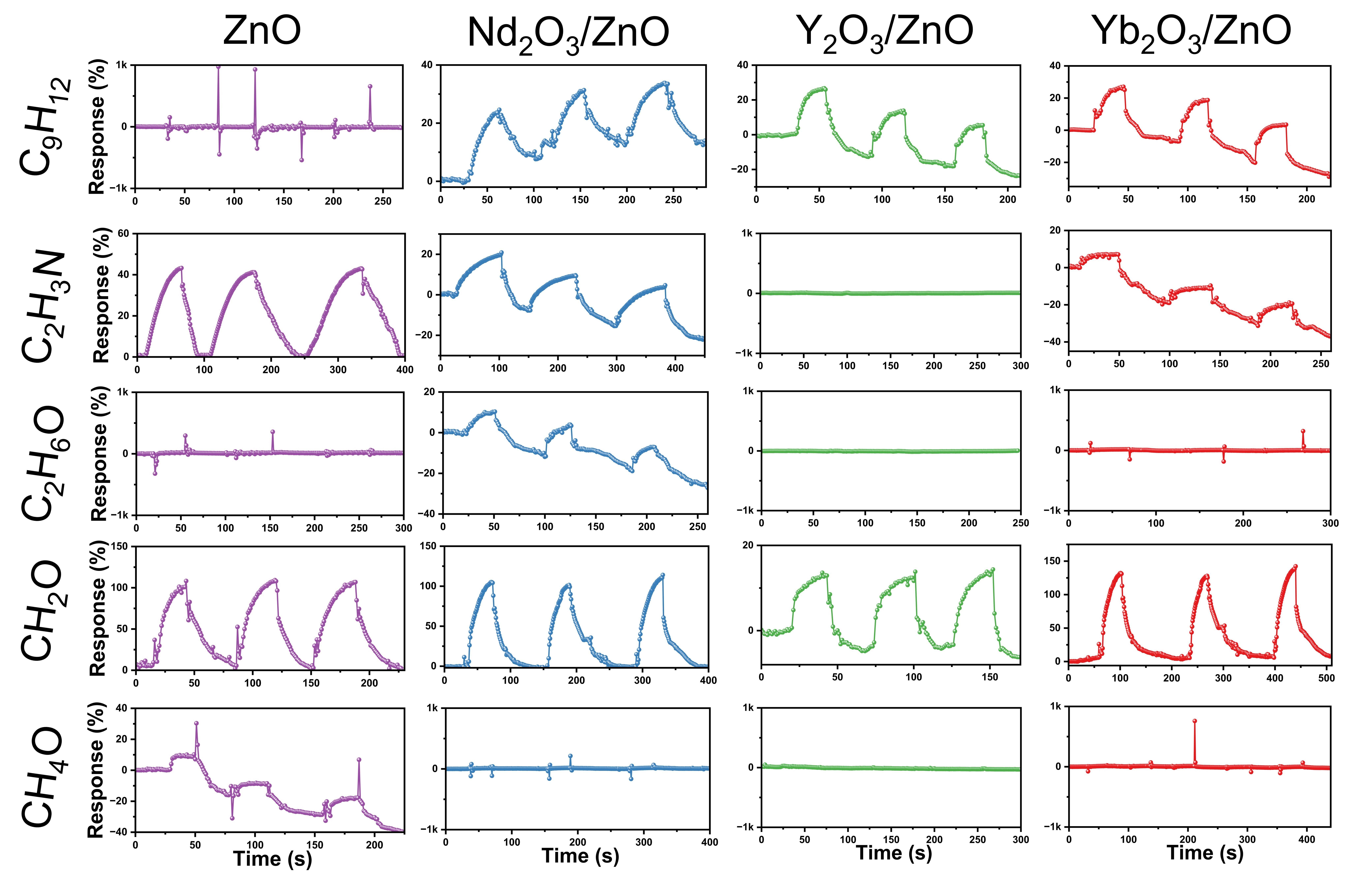


**Figure S10.** Dynamic response curves of ZnO and RE_2_O_3_/ZnO (RE=Nd, Y, Yb) to 500 ppm of C_9_H_12_, C_2_H_3_N, C_2_H_5_OH, CH_2_O, and CH_3_OH gases under RT conditions.


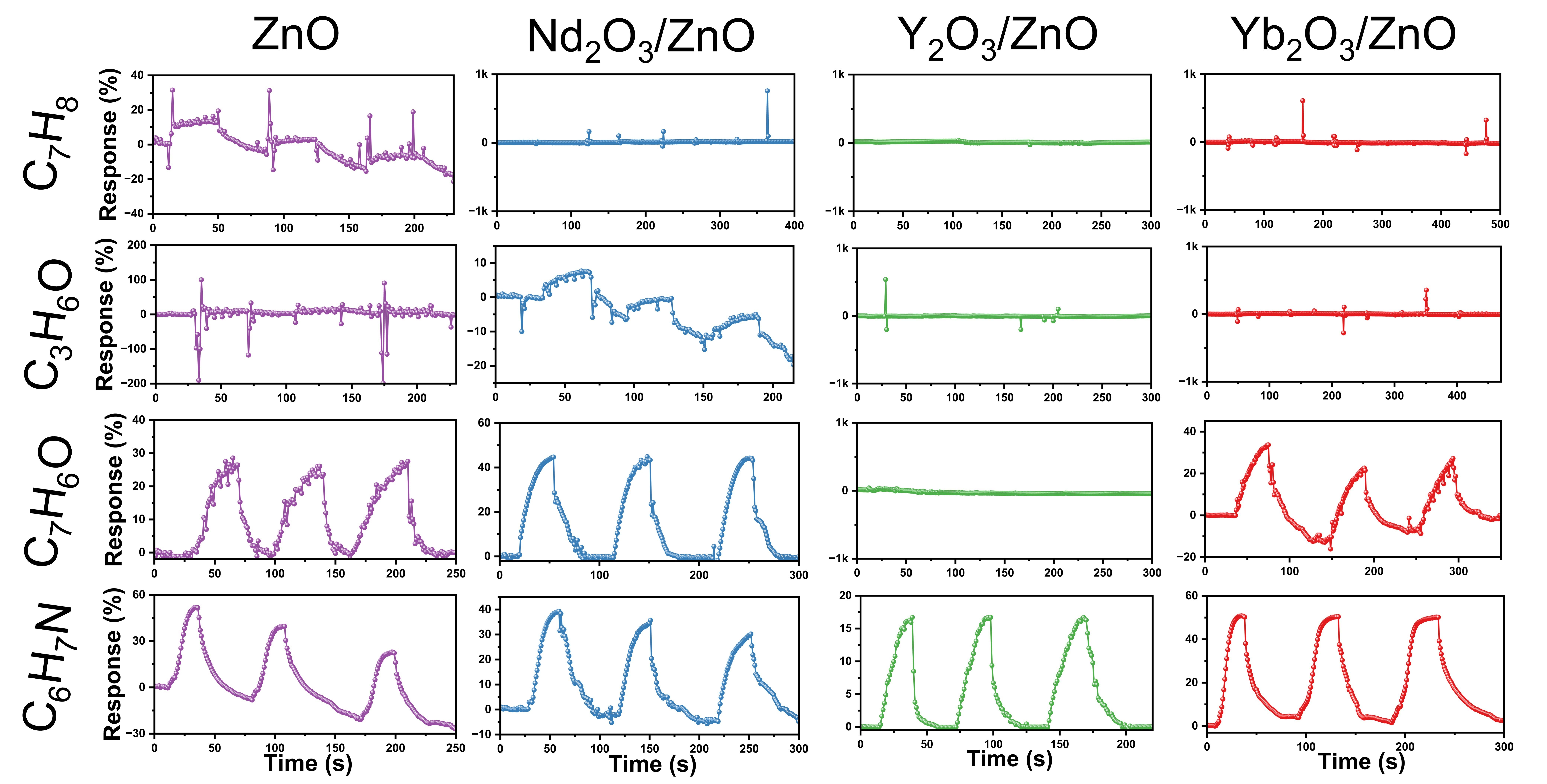


**Figure S11.** Dynamic response curves of ZnO and RE_2_O_3_/ZnO (RE=Nd, Y, Yb) to 500 ppm of C_7_H_8_, C_3_H_6_O, C_7_H_6_O, and C_6_H_7_N gases under RT conditions.


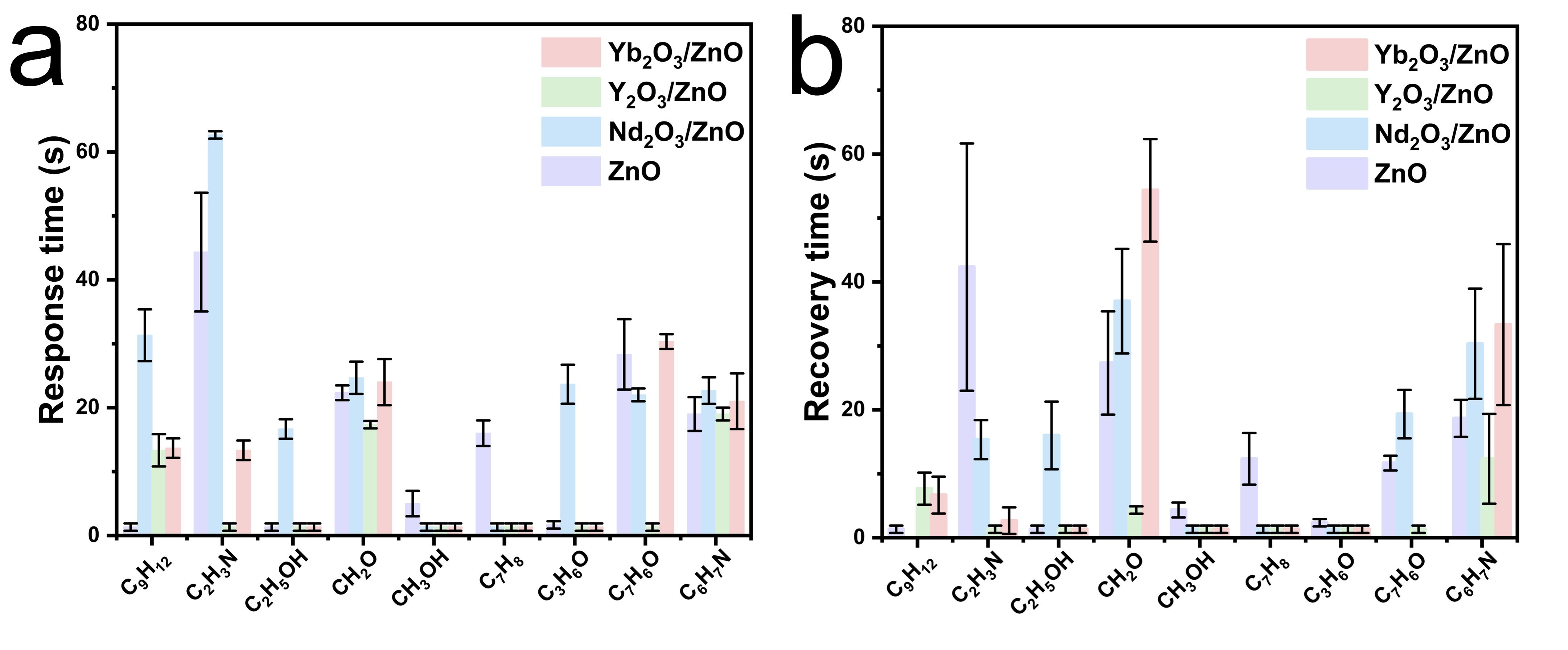


**Figure S12.** Response time a) and recovery time b) of ZnO and RE_2_O_3_/ZnO (RE=Nd, Y, Yb) to 500 ppm of C_9_H_12_, C_2_H_3_N, C_2_H_5_OH, CH_2_O, CH_3_OH, C_7_H_8_, C_3_H_6_O, C_7_H_6_O and C_6_H_7_N gases under RT conditions.


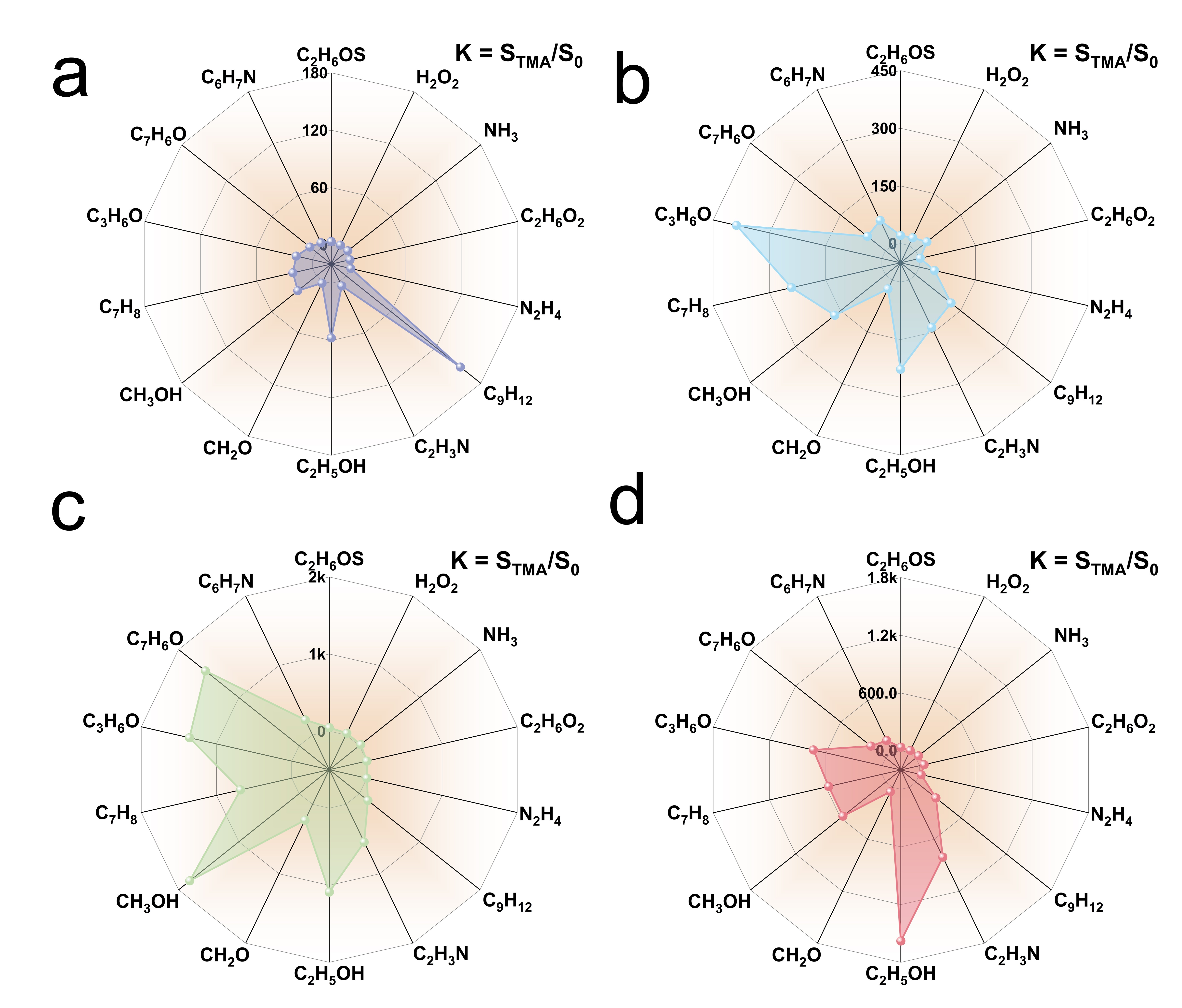


**Figure S13.** The cross-selectivity profiles of a) ZnO, b) Nd_2_O_3_/ZnO, c) Y_2_O_3_/ZnO, and d) Yb_2_O_3_/ZnO toward interfering gases, namely C_2_H_6_OS, H_2_O_2_, NH_3_, C_2_H_6_O_2_, N_2_H_4_, C_9_H_12_, C_2_H_3_N, C_2_H_5_OH, CH_2_O, CH_3_OH, C_7_H_8_, C_3_H_6_O, C_7_H_6_O, and C_6_H_7_N.


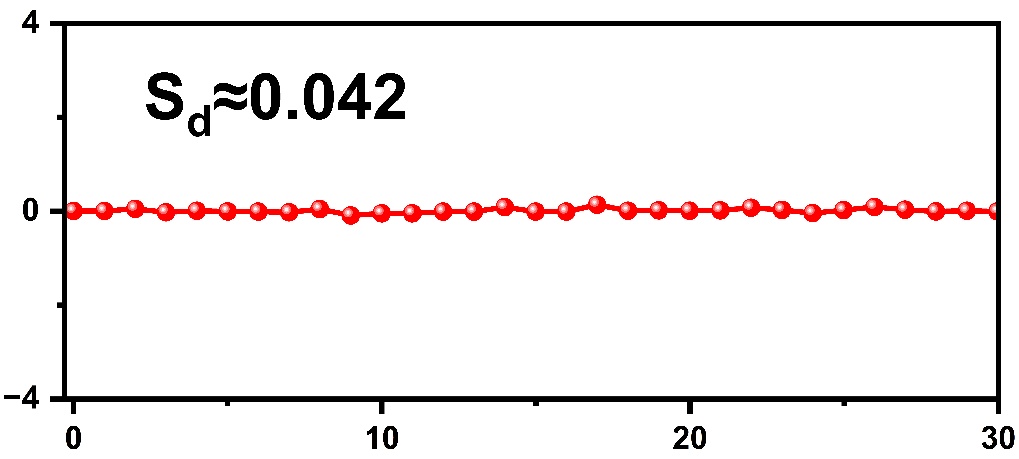


**Figure S14.** Response curve noise of Yb_2_O_3_/ZnO sensor.


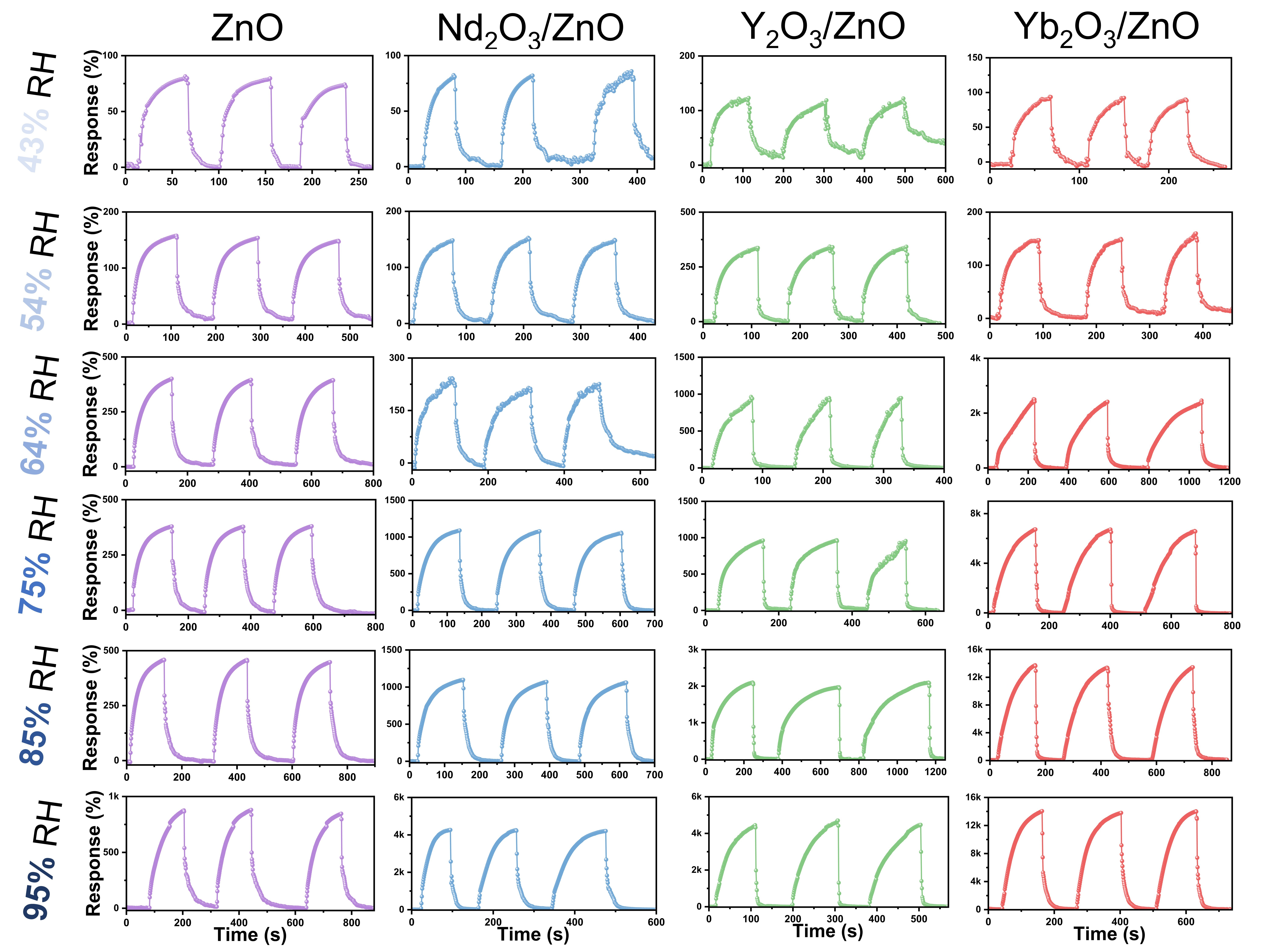


**Figure S15.** Dynamic response curves of ZnO and RE_2_O_3_/ZnO sensors under different humidity conditions.


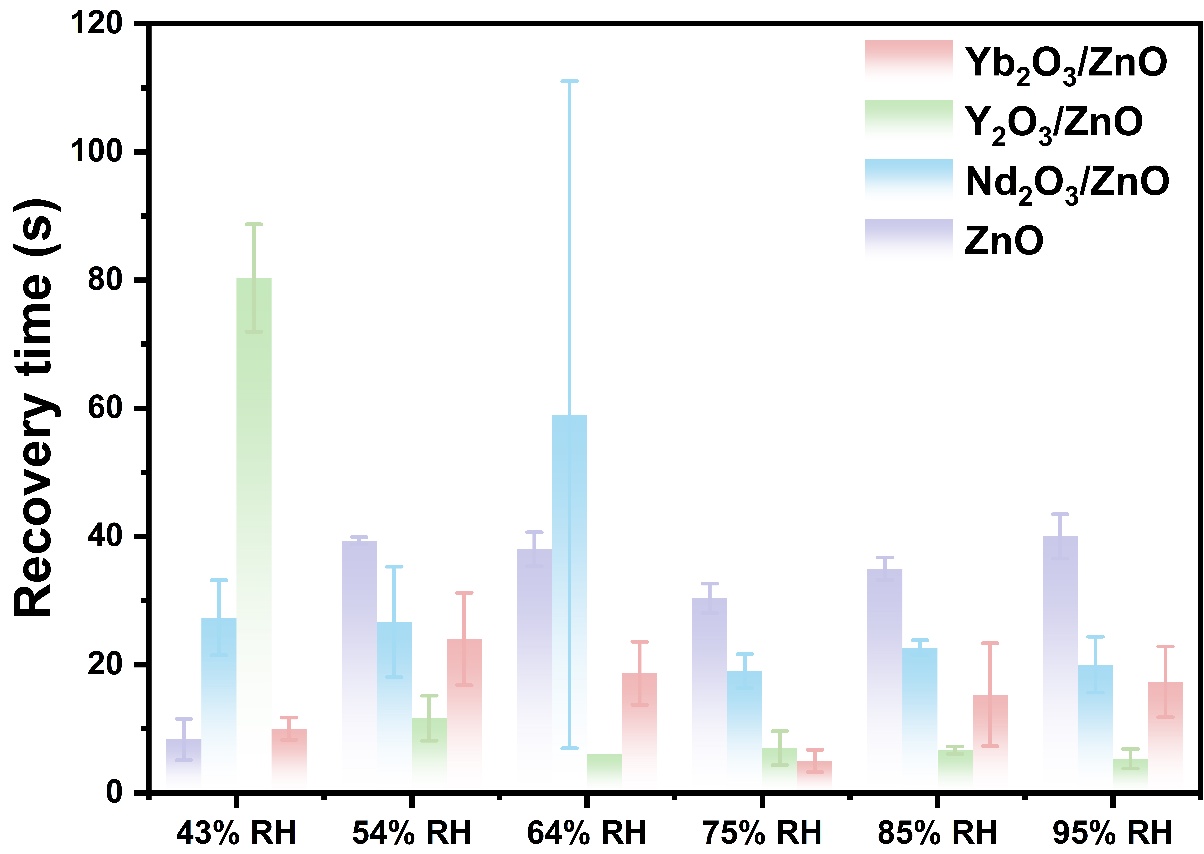


**Figure S16.** Recovery time of ZnO and RE_2_O_3_/ZnO sensors under different humidity conditions.


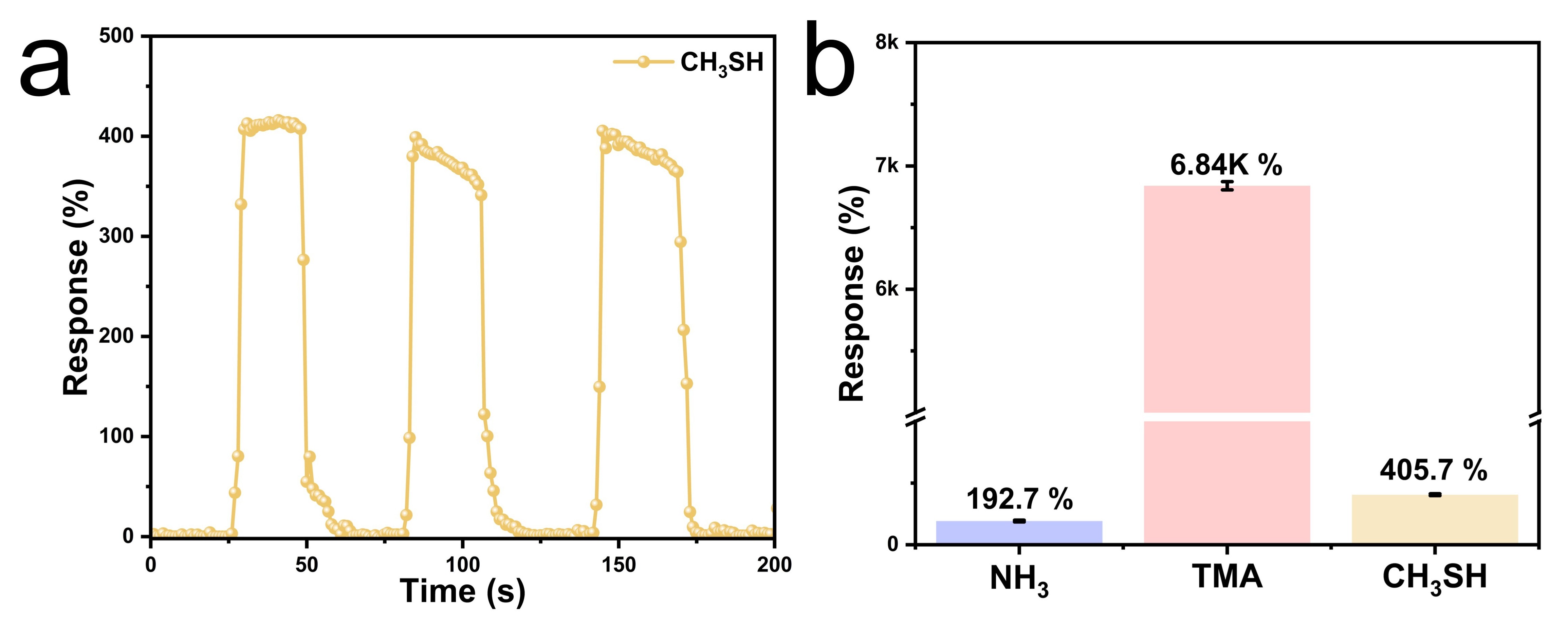


**Figure S17.** Yb_2_O_3_/ZnO sensor response curves at room temperature for 500 ppm a) CH_3_SH and b) response comparison values (NH_3_, TMA, CH_3_SH).

Although humidity contributes to the overall sensing signal to some extent, the dominant response originates from spoilage gases. As shown in Figure S18, under equivalent relative humidity conditions, the sensor response in actual spoilage environments consistently and significantly exceeds that in humidity-only conditions, indicating that the signal enhancement caused by spoilage gases far surpasses the effect of humidity alone. Meanwhile, the influence of humidity on response kinetics primarily manifests as baseline resistance drift through physisorption, whereas spoilage gases generate responses through specific chemical interactions with the sensing material. The distinct differences in their response patterns further confirm the dominant role of spoilage metabolites.





**Figure S18.** Comparison of humidity response at RT and response to fish meat spoilage products from 0 to 52 h in Yb_2_O_3_/ZnO sensors.


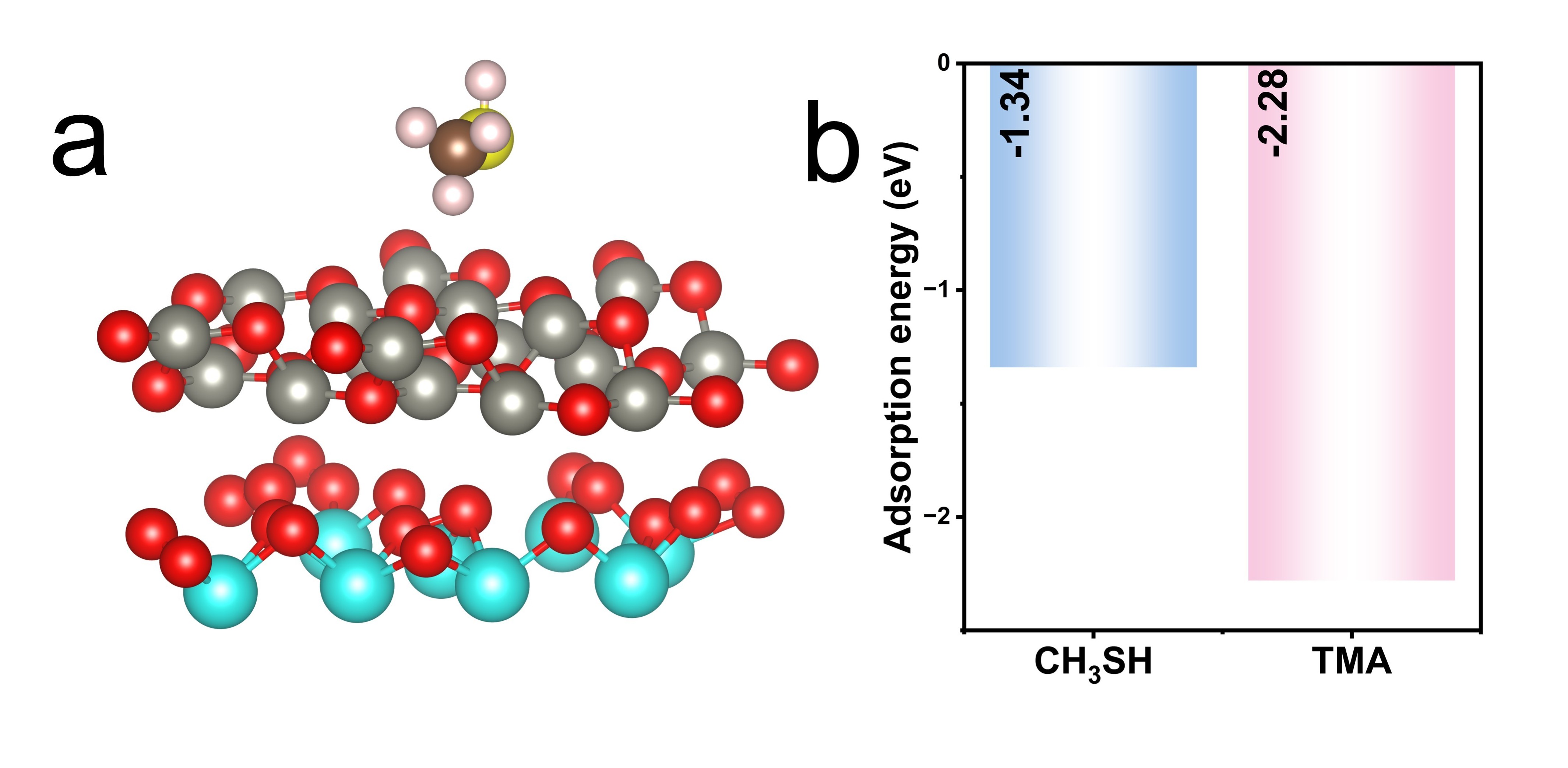


**Figure S19.** a) Optimized structure of CH_3_SH adsorbed on Yb_2_O_3_/ZnO; b) Comparison of adsorption energies for CH_3_SH and TMA on Yb_2_O_3_/ZnO.


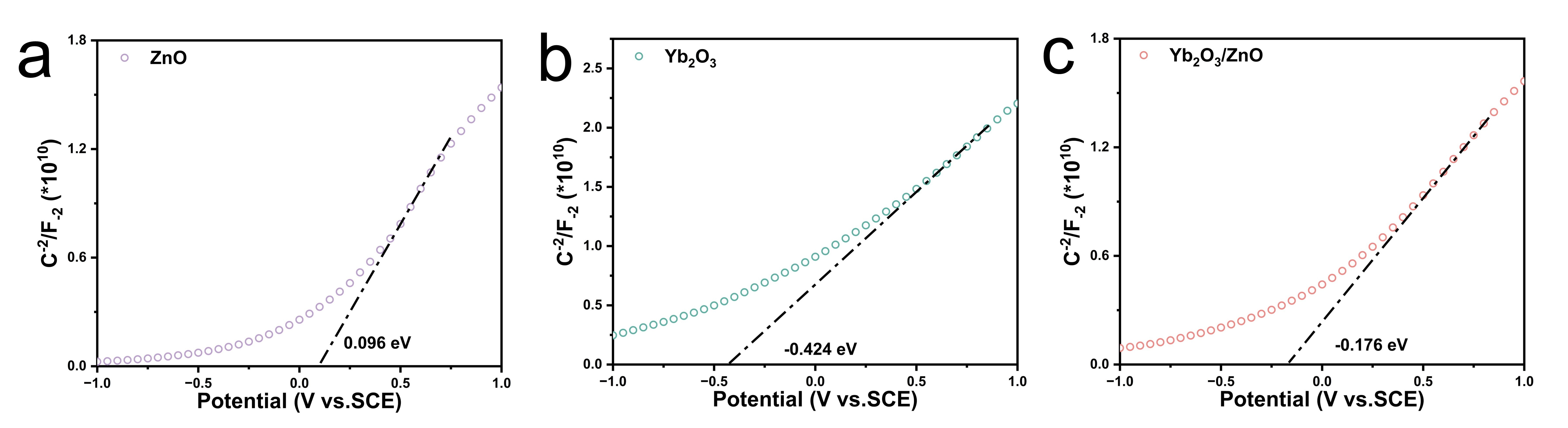


**Figure S20.** Mott-Schottky curves of a) ZnO, b) Yb_2_O_3_, and c) Yb_2_O_3_/ZnO samples.


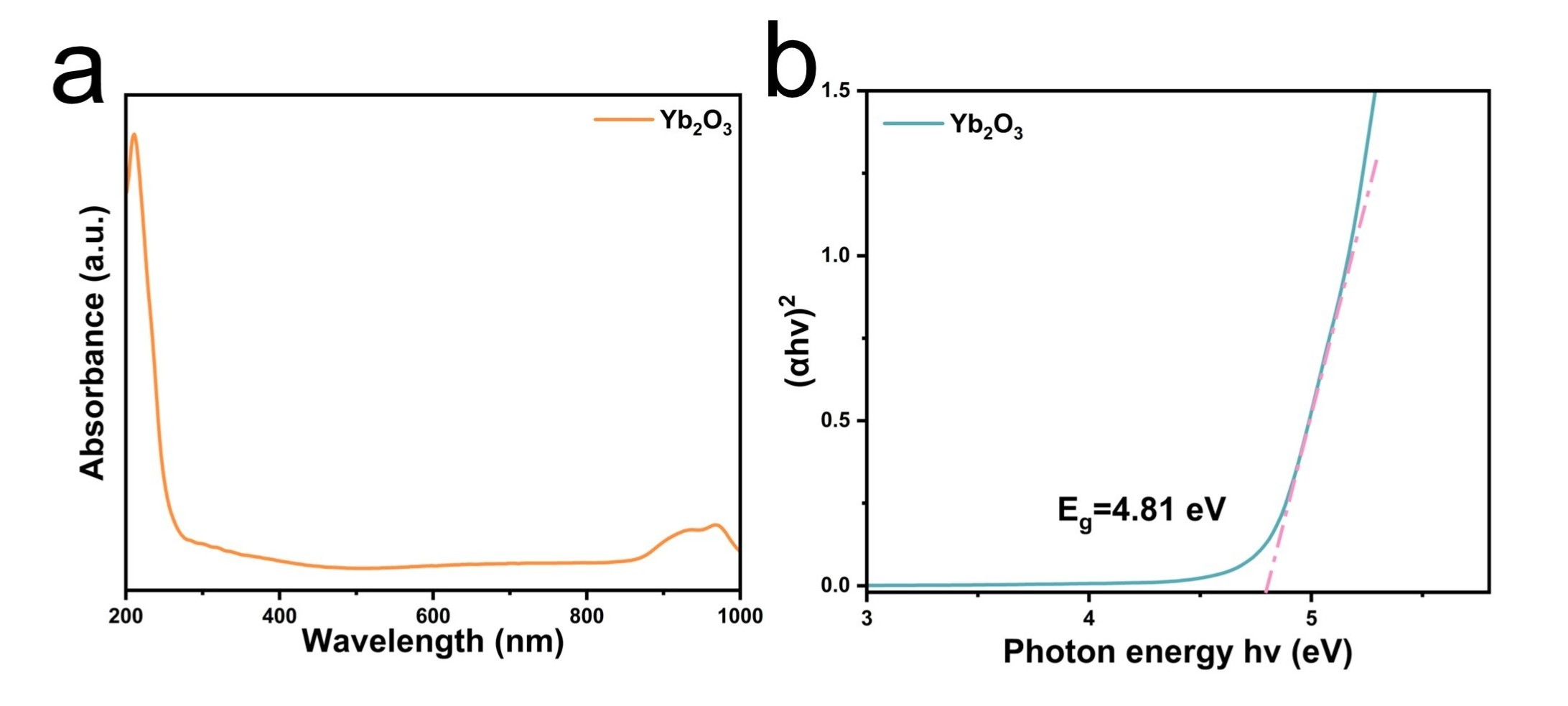


**Figure S21.** a) UV-Vis and b) Tauc spectra of Yb_2_O_3_.


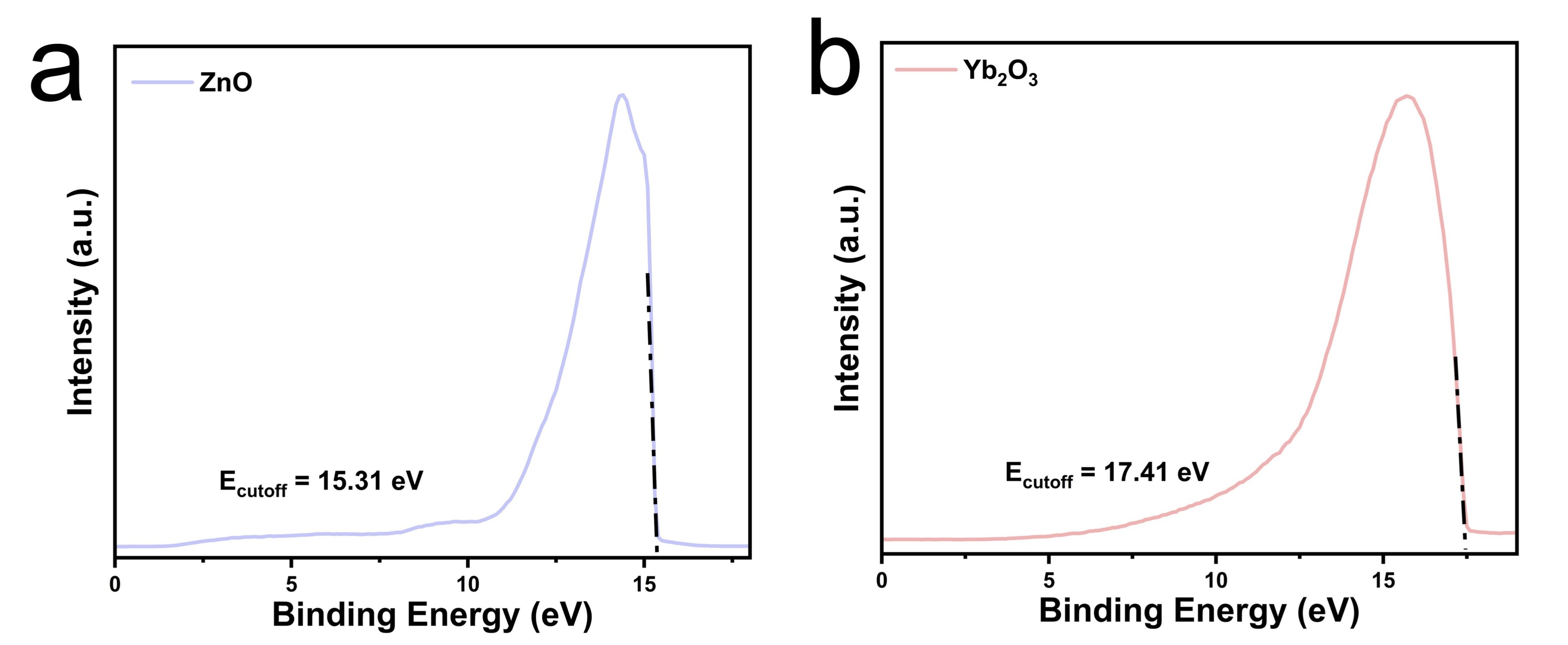


**Figure S22.** UPS spectrum of a) ZnO and b) Yb_2_O_3_.


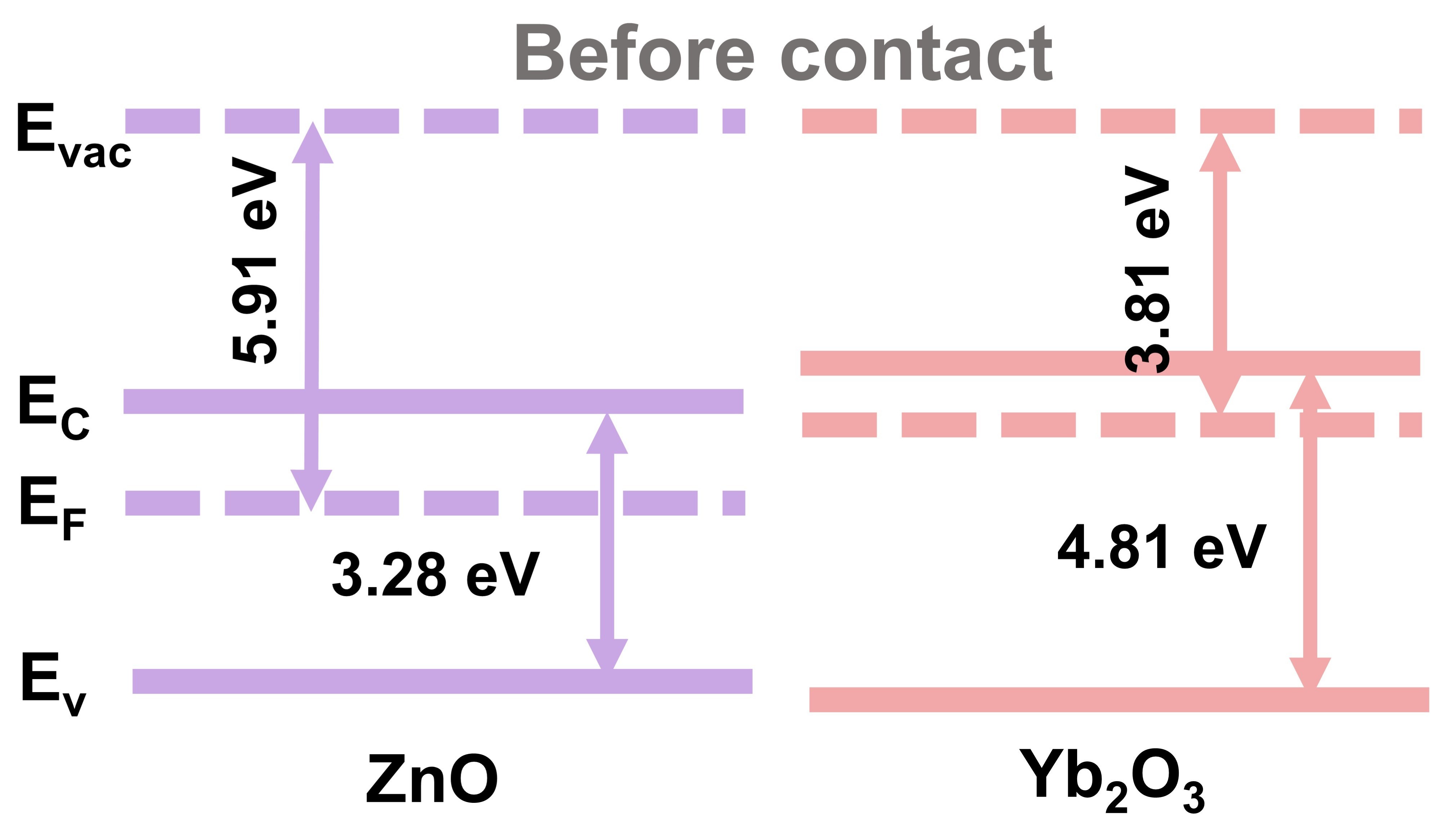


**Figure S23.** Energy band structures of the individual oxides (ZnO, Yb_2_O_3_).


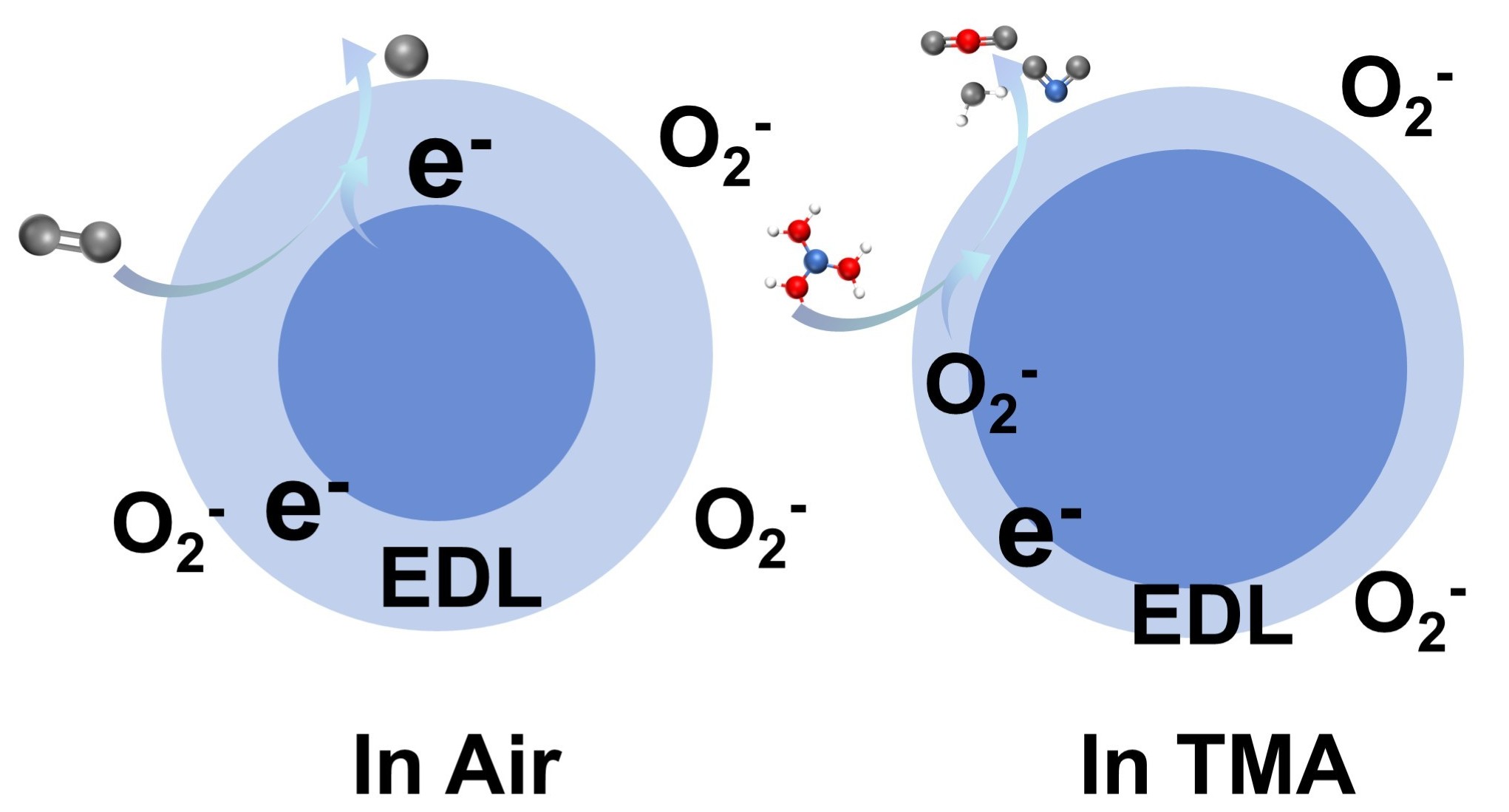


**Figure S24.** Yb_2_O_3_/ZnO EDL Change Images in air and in TMA.





**Figure S25.** KPFM analysis of ZnO and Yb_2_O_3_/ZnO. a, c) AFM and 2D KPFM images of ZnO and Yb_2_O_3_/ZnO before exposure. b, d) AFM and 2D KPFM images of ZnO and Yb_2_O_3_/ZnO after exposure. e) CPD values of ZnO and Yb_2_O_3_/ZnO before and after exposure; f, g) pixel histograms of CPD.


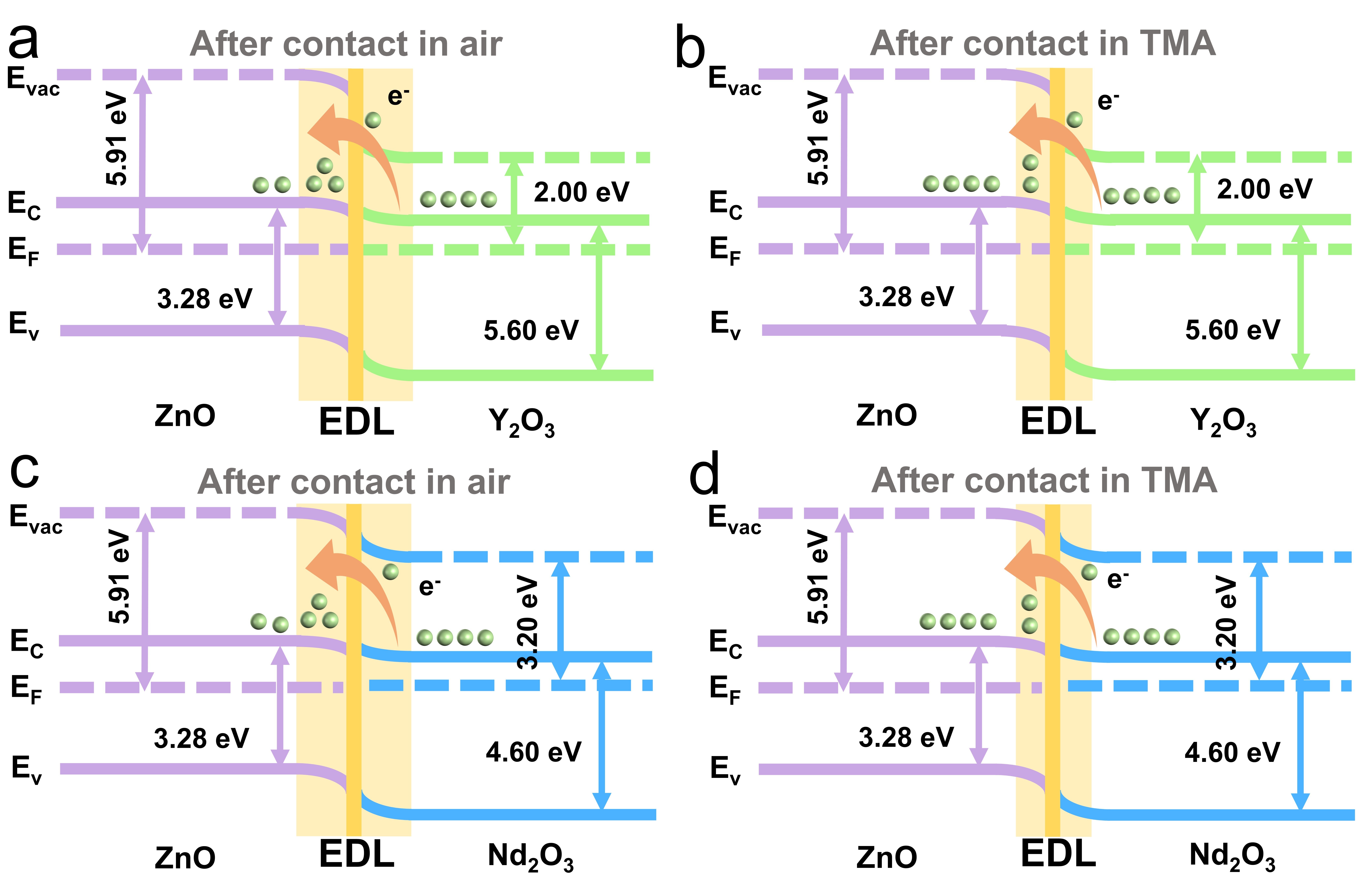


**Figure S26.** a-b) Y_2_O_3_/ZnO and c-d) Nd_2_O_3_/ZnO: energy band diagrams in air and in TMA.


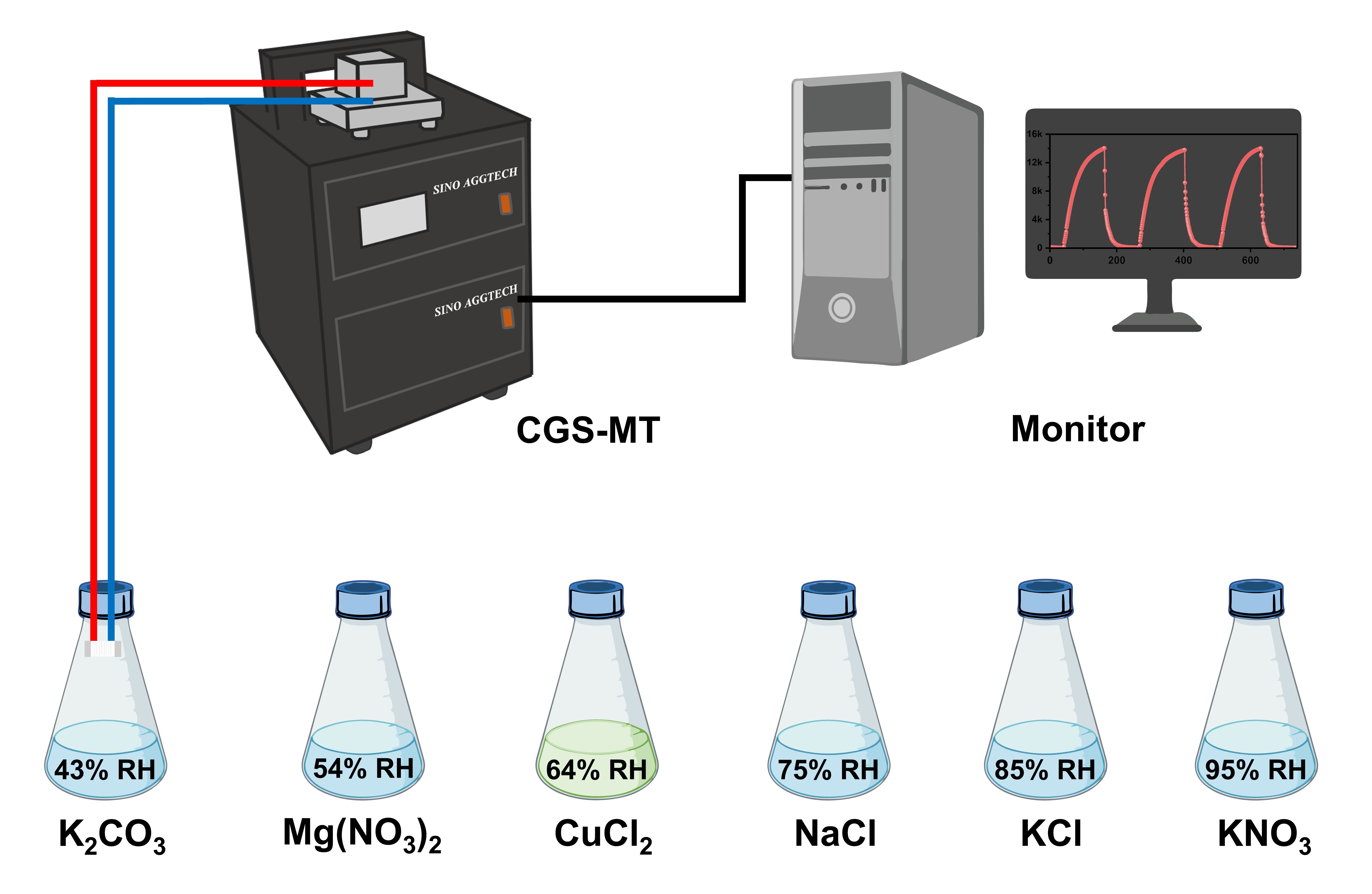


**Figure S27.** Schematic diagram of the humidity testing process.

**Table S1.** Specific LoD data for the Yb_2_O_3_/ZnO sensor and other TMA sensors

| Sensing materials | Temperature (℃) | LoD (ppm) | Year of publication | Ref. |
| --- | --- | --- | --- | --- |
| Ru-SnO_2_ | 400 | 1 | 2012 | [7] |
| Au/WO_3_ | 300 | 0.5 | 2022 | [8] |
| Au-MoO_3_ | 280 | 5 | 2016 | [9] |
| In_2_O_3_-SnO_2_ NFs | 280 | 1 | 2016 | [10] |
| α-Fe_2_O_3_/TiO_2_ | 250 | 10 | 2013 | [11] |
| 5%Ga-In_2_O_3_ NTs | 240 | 0.5 | 2022 | [12] |
| α-Fe_2_O_3_ | 217 | 0.1 | 2021 | [13] |
| Co_3_O_4_/In_2_O_3_ | 200 | 1 | 2023 | [14] |
| NiO/In_2_O_3_ | 200 | 0.5 | 2021 | [15] |
| Co_3_O_4_/SnO_2_ | 175 | 1 | 2020 | [16] |
| LIG/MoS | 100 | 0.0027 | 2022 | [17] |
| In_2_O_3_ | 80 | 1 | 2020 | [18] |
| [Co(im)_2_]_n_ | 75 | 2 | 2014 | [19] |
| WO_3_ | 70 | 50 | 2001 | [20] |
| GO/Cs | 25 | 1.3 | 2016 | [21] |
| PAA - NaCl CNM | 25 | 1 | 2010 | [22] |
| V_2_O_3_-Cu_2_O | 25 | 3 | 2019 | [23] |
| PANI/TiO_2_ | 25 | 10 | 2008 | [24] |
| PANI | 25 | 5 | 2007 | [25] |
| SnS_X_-6 | 25 | 0.77 | 2022 | [26] |
| E-g-C_3_N_4_/Bi_2_MoO_6_ | 22 | 1.3 | 2023 | [27] |
| Yb_2_O_3_/ZnO | 25 | 0.919 | - | This work |

**Table S2.** Dipole moment, key chemical bonds, bond strength, and ionization energy values of TMA and VOCs gases.

| Compound | Dipole moment (Debye) | Key chemical bonds | Bond strength (kJ/mol)/ Ionization energy (eV) | Ref. |
| --- | --- | --- | --- | --- |
| C_2_H_3_N | 3.92^a^ | C≡N | 891^b^ / 12.20^c^ | [28]a [29]b [30]c |
| C_6_H_7_N | 1.71^a^ | C–N | 307^b^ / 8.10^c^ | [31]a [32]b [33]c |
| CH_2_O | 2.33^a^ | C=O | 747^b^ / 10.88^c^ | [34]a [35]b [36]c |
| C_2_H_6_OS | 3.96^a^ | S=O | 522^b^ / 9.10^c^ | [37]a [38]b [39]c |
| NH_3_ | 1.94^a^ | N–H | 386^b^ /10.02^c^ | [40]a [38]b [41]c |
| C_3_H_9_N (TMA) | 0.87^a^ | C–N | 307^b^ / 7.82^c^ | [42]a [32]b [43]c |

**Table S3.** Parameters required for testing gas concentration.

| RN | B | PD | P | MWS | CGFR | GC | LPR |
| --- | --- | --- | --- | --- | --- | --- | --- |
| C_2_H_6_O_2_ | 197.3 | 1.11 | 99.00 | 62.07 | 1000 | 1000 | 1257.3600 |
| C_3_H_6_O | 56 | 0.79 | 99.50 | 58.08 | 1000 | 1000 | 3306.9627 |
| NH_3_ | 38 | 0.91 | 25.00 | 17.03 | 1000 | 1000 | 3341.8367 |
| C_2_H_6_O | 78.5 | 0.79 | 99.70 | 46.07 | 1000 | 1000 | 2611.2469 |
| C_2_H_3_N | 81.6 | 0.786 | 99.90 | 41.05 | 1000 | 1000 | 5789.2347 |
| N_2_H_4_ | 113.5 | 1.011 | 50-60 | 32.0452 | 1000 | 1000 | 1670.9180 |
| C_3_H_9_N | 26 | 0.90 | 33.00 | 59.11 | 1000 | 1000 | 8884.9808 |
| C_2_H_6_OS | 189 | 1.10 | 99.50 | 78.13 | 1000 | 1000 | 3189.2448 |
| C_9_H_12_ | 152 | 0.86 | 99.50 | 120.19 | 1000 | 1000 | 6282.4658 |
| C_7_H_8_ | 110.6 | 0.87 | 99.50 | 92.14 | 1000 | 1000 | 4757.2649 |
| H_2_O_2_ | 158 | 1.13 | 99.90 | 34.01 | 1000 | 1000 | 1344.9765 |
| C_7_H_6_O | 179 | 1.04 | 99.00 | 106.12 | 1000 | 1000 | 2400.0000 |
| C_6_H_7_N | 184 | 1.02 | 99.50 | 93.13 | 1000 | 1000 | 4088.5339 |

Reagent Name (**RN**)

Boiling (**B**)

Point Density (g cm^-3^) (**PD**)

Purity (%) (**P**)

Molecular Weight of Substance M (g mol^-1^) (**MWS**)

Carrier Gas Flow Rate (mL min^-1^) (**CGFR**)

Gas Concentration (ppm) (**GC**)

Liquid Propulsion Rate (nL min^-1^) (**LPR**)

**Reference**

1. C. Yang, H. Zhang, "Preparation and performance study of humidity sensor based on defect-controlled TiO_2_/CdS heterostructure," *Sensors and Actuators B: Chemical* 404 (2024): 135321, https://doi.org/10.1016/j.snb.2024.135321.

2. S. Yu, C. Chen, H. Zhang, et al., "Design of high sensitivity graphite carbon nitride/zinc oxide humidity sensor for breath detection," *Sensors and Actuators B: Chemical* 332 (2021): 129536, https://doi.org/10.1016/j.snb.2021.129536.

3. G. Kresse, J. Furthmüller, "Efficiency of ab-initio total energy calculations for metals and semiconductors using a plane-wave basis set," *Computational Materials Science* 6 (1996): 15-50, https://doi.org/10.1016/0927-0256(96)00008-0.

4. S. Zeb, Z. Yang, R. Hu, et al., "Electronic structure and oxygen vacancy tuning of Co & Ni co-doped W_18_O_49_ nanourchins for efficient TEA gas sensing," *Chemical Engineering Journal* 465 (2023): 142815, https://doi.org/10.1016/j.cej.2023.142815.

5. S. Grimme, J. Antony, S. Ehrlich, et al., "A consistent and accurate ab initio parametrization of density functional dispersion correction (DFT-D) for the 94 elements H-Pu," *The Journal of Chemical Physics* 132 (2010), https://doi.org/10.1063/1.3382344.

6. V. Wang, N. Xu, J.-C. Liu, et al., "VASPKIT: A user-friendly interface facilitating high-throughput computing and analysis using VASP code," *Computer Physics Communications* 267 (2021): 108033, https://doi.org/10.1016/j.cpc.2021.108033.

7. K.-M. Kim, K.-I. Choi, H.-M. Jeong, et al., "Highly sensitive and selective trimethylamine sensors using Ru-doped SnO_2_ hollow spheres," *Sensors and Actuators B: Chemical* 166-167 (2012): 733-738, https://doi.org/10.1016/j.snb.2012.03.049.

8. C. Zhao, J. Shen, S. Xu, et al., "Ultra-efficient trimethylamine gas sensor based on Au nanoparticles sensitized WO_3_ nanosheets for rapid assessment of seafood freshness," *Food Chemistry* 392 (2022): 133318, https://doi.org/10.1016/j.foodchem.2022.133318.

9. J. Zhang, P. Song, Z. Li, et al., "Enhanced trimethylamine sensing performance of single-crystal MoO_3_ nanobelts decorated with Au nanoparticles," *Journal of Alloys and Compounds* 685 (2016): 1024-1033, https://doi.org/10.1016/j.jallcom.2016.06.257.

10. F. Li, X. Gao, R. Wang, et al., "Design of Core–Shell Heterostructure Nanofibers with Different Work Function and Their Sensing Properties to Trimethylamine," *ACS Applied Materials & Interfaces* 8 (2016): 19799-19806, https://doi.org/10.1021/acsami.6b04063.

11. Z. Lou, F. Li, J. Deng, et al., "Branch-like Hierarchical Heterostructure (α-Fe_2_O_3_/TiO_2_): A Novel Sensing Material for Trimethylamine Gas Sensor," *ACS Applied Materials & Interfaces* 5 (2013): 12310-12316, https://doi.org/10.1021/am402532v.

12. W. Ren, C. Zhao, G. Niu, et al., "Gas Sensor Array with Pattern Recognition Algorithms for Highly Sensitive and Selective Discrimination of Trimethylamine," *Advanced Intelligent Systems* 4 (2022): 2200169, https://doi.org/10.1002/aisy.202200169.

13. P. Wang, L. Sui, H. Yu, et al., "Monodispersed hollow α-Fe_2_O_3_ ellipsoids via [C_12_mim][PF_6_]-assistant synthesis and their excellent n-butanol gas-sensing properties," *Sensors and Actuators B: Chemical* 326 (2021): 128796, https://doi.org/10.1016/j.snb.2020.128796.

14. Y. Ji, N. Zhang, J. Xu, et al., "Co_3_O_4_/In_2_O_3_ p-n heterostructures based gas sensor for efficient structure-driven trimethylamine detection," *Ceramics International* 49 (2023): 17354-17362, https://doi.org/10.1016/j.ceramint.2023.02.103.

15. D. Meng, T. Qiao, G. Wang, et al., "NiO-functionalized In_2_O_3_ flower-like structures with enhanced trimethylamine gas sensing performance," *Applied Surface Science* 577 (2022): 151877, https://doi.org/10.1016/j.apsusc.2021.151877.

16. D. Meng, J. Si, M. Wang, et al., "One-step synthesis and the enhanced trimethylamine sensing properties of Co_3_O_4_/SnO_2_ flower-like structures," *Vacuum* 171 (2020): 108994, https://doi.org/10.1016/j.vacuum.2019.108994.

17. J. Zhao, N. Yi, X. Ding, et al., "In situ laser-assisted synthesis and patterning of graphene foam composites as a flexible gas sensing platform," *Chemical Engineering Journal* 456 (2023): 140956, https://doi.org/10.1016/j.cej.2022.140956.

18. Y. Li, J. Liu, J. Zhang, et al., "Deposition of In_2_O_3_ nanofibers on polyimide substrates to construct high-performance and flexible trimethylamine sensor," *Chinese Chemical Letters* 31 (2020): 2142-2144, https://doi.org/10.1016/j.cclet.2019.11.048.

19. E.-X. Chen, H.-R. Fu, R. Lin, et al., "Highly Selective and Sensitive Trimethylamine Gas Sensor Based on Cobalt Imidazolate Framework Material," *ACS Applied Materials & Interfaces* 6 (2014): 22871-22875, https://doi.org/10.1021/am5071317.

20. M. Tong, G. Dai, D. Gao, "WO_3_ thin film sensor prepared by sol–gel technique and its low-temperature sensing properties to trimethylamine," *Materials Chemistry and Physics* 69 (2001): 176-179, https://doi.org/10.1016/S0254-0584(00)00389-8.

21. K. Zhang, R. Hu, G. Fan, et al., "Graphene oxide/chitosan nanocomposite coated quartz crystal microbalance sensor for detection of amine vapors," *Sensors and Actuators B: Chemical* 243 (2017): 721-730, https://doi.org/10.1016/j.snb.2016.12.063.

22. X. Wang, B. Ding, J. Yu, et al., "Electro-netting: Fabrication of two-dimensional nano-nets for highly sensitive trimethylamine sensing," *Nanoscale* 3 (2011): 911-915, http://dx.doi.org/10.1039/C0NR00783H.

23. V. Mounasamy, G. K. Mani, D. Ponnusamy, et al., "Sub-ppm level detection of trimethylamine using V_2_O_3_-Cu_2_O mixed oxide thin films," *Ceramics International* 45 (2019): 19528-19533, https://doi.org/10.1016/j.ceramint.2019.06.074.

24. J. Zheng, G. Li, X. Ma, et al., "Polyaniline–TiO_2_ nano-composite-based trimethylamine QCM sensor and its thermal behavior studies," *Sensors and Actuators B: Chemical* 133 (2008): 374-380, https://doi.org/10.1016/j.snb.2008.02.037.

25. G. Li, J. Zheng, X. Ma, et al., in *Sensors, Vol. 7*, 2007, pp. 2378-2388.

26. Q. a. Zhou, C. Zheng, L. Zhu, et al., "Tin sulfides heterostructure modified quartz crystal microbalance sensors with high sensitivity for hazardous trimethylamine gas," *Sensors and Actuators B: Chemical* 371 (2022): 132520, https://doi.org/10.1016/j.snb.2022.132520.

27. K. Wu, X. He, A. Ly, et al., "Highly sensitive and selective gas sensors based on 2D/3D Bi_2_MoO_6_ micro-nano composites for trimethylamine biomarker detection," *Applied Surface Science* 629 (2023): 157443, https://doi.org/10.1016/j.apsusc.2023.157443.

28. E. M. Cabaleiro-Lago, J. M. Hermida-Ramón, A. Peña-Gallego, et al., "Intermolecular interactions and cooperative effects in acetonitrile clusters. An ab initio molecular orbital study," *Journal of Molecular Structure: THEOCHEM* 498 (2000): 21-28, https://doi.org/10.1016/S0166-1280(99)00207-9.

29. R. T. Sanderson, "Electronegativity and bond energy," *Journal of the American Chemical Society* 105 (1983): 2259-2261, https://doi.org/10.1021/ja00346a026.

30. M. Gochel-Dupuis, J. Delwiche, M. J. Hubin-Franskin, et al., "High-resolution HeI photoelectron spectrum of acetonitrile," *Chemical Physics Letters* 193 (1992): 41-48, https://doi.org/10.1016/0009-2614(92)85680-9.

31. M. Targema, N. O. Obi-Egbedi, M. D. Adeoye, "Molecular structure and solvent effects on the dipole moments and polarizabilities of some aniline derivatives," *Computational and Theoretical Chemistry* 1012 (2013): 47-53, https://doi.org/10.1016/j.comptc.2013.02.020.

32. V. Mounasamy, G. K. Mani, D. Ponnusamy, et al., "Network mixed metal oxide (V^4+^ and Ti^4+^) nanostructures as potential material for the detection of trimethylamine," *New Journal of Chemistry* 43 (2019): 11069-11081, http://dx.doi.org/10.1039/C9NJ00727J.

33. H. J. Haink, J. E. Adams, J. R. Huber, "The Electronic Structure of Aromatic Amines: Photoelectron Spectroscopy of Diphenylamine, Iminobibenzyl, Acridan and Carbazole," *Berichte der Bunsengesellschaft für physikalische Chemie* 78 (1974): 436-440, https://doi.org/10.1002/bbpc.19740780503.

34. F. J. Lovas, C. L. Lugez, "The Microwave Spectrum and Structure of CH_2_O–H_2_O," *Journal of Molecular Spectroscopy* 179 (1996): 320-323, https://doi.org/10.1006/jmsp.1996.0210.

35. T. Ravikumar, L. Thirumalaisamy, S. Madanagurusamy, et al., "Manganese doped two-dimensional zinc ferrite thin films as chemiresistive trimethylamine gas sensors," *Physical Chemistry Chemical Physics* 25 (2023): 32216-32233, http://dx.doi.org/10.1039/D3CP03867J.

36. K. Ohno, K. Okamura, H. Yamakado, et al., "Penning Ionization of HCHO, CH_2_CH_2_, and CH_2_CHCHO by Collision with He(2^3^S) Metastable Atoms," *The Journal of Physical Chemistry* 99 (1995): 14247-14253, https://doi.org/10.1021/j100039a010.

37. C. Di Mino, A. J. Clancy, A. Sella, et al., "Weak Interactions in Dimethyl Sulfoxide (DMSO)–Tertiary Amide Solutions: The Versatility of DMSO as a Solvent," *The Journal of Physical Chemistry B* 127 (2023): 1357-1366, https://doi.org/10.1021/acs.jpcb.2c07155.

38. B. d. B. Darwent, in *National Bureau of Standards,* Vol. 31, 1970, pp. 15-22.

39. K. Kimura, *Handbook of HeI photoelectron spectra of fundamental organic molecules: ionization energies, ab initio assignments, and valence electronic structure for 200 molecules*, Japan scientific societies Press, 1981.

40. T. A. R. Irwin, F. W. Dalby, "EXPERIMENTAL DETERMINATION OF THE DIPOLE MOMENTS OF THE DEGENERATE STATES OF NH," *Canadian Journal of Physics* 43 (1965): 1766-1775, https://cdnsciencepub.com/doi/abs/10.1139/p65-171.

41. F. Qi, L. Sheng, Y. Zhang, et al., "Experimental and theoretical study of the dissociation energies D_0_(H_2_N H) and D0(H_2_N^+^ H) and other related quantities," *Chemical Physics Letters* 234 (1995): 450-454, https://doi.org/10.1016/0009-2614(95)00059-D.

42. P. B. Ryan, H. D. Todd, "Explicit representation of lone‐pair orbitals in molecular orbital calculations for NH_3_, H_2_O, N_2_H_4_, and H_2_O_2_," *Journal of Chemical Physics* 67 (1977): 4787-4793, https://doi.org/10.1063/1.434682.

43. M. Meot-Ner, S. F. Nelsen, M. F. Willi, et al., "Special effects of an unusually large neutral to radical cation geometry change. Adiabatic ionization energies and proton affinities of alkylhydrazines," *Journal of the American Chemical Society* 106 (1984): 7384-7389, https://doi.org/10.1021/ja00336a015.
